# Molecular basis of the microtubule-regulating activity of microtubule crosslinking factor 1

**DOI:** 10.1371/journal.pone.0182641

**Published:** 2017-08-07

**Authors:** Mohammad Abdul Kader, Tomoko Satake, Masatoshi Yoshida, Ikuko Hayashi, Atsushi Suzuki

**Affiliations:** 1 Molecular Cellular Biology Laboratory, Yokohama City University Graduate School of Medical Life Science, Tsurumi-ku, Yokohama, Japan; 2 Molecular Medical Bioscience Laboratory, Yokohama City University Graduate School of Medical Life Science, Tsurumi-ku, Yokohama, Japan; Institut de Genetique et Developpement de Rennes, FRANCE

## Abstract

The variety of microtubule arrays observed across different cell types should require a diverse group of proteins that control microtubule organization. Nevertheless, mainly because of the intrinsic propensity of microtubules to easily form bundles upon stabilization, only a small number of microtubule crosslinking proteins have been identified, especially in postmitotic cells. Among them is microtubule crosslinking factor 1 (MTCL1) that not only interconnects microtubules via its N-terminal microtubule-binding domain (N-MTBD), but also stabilizes microtubules via its C-terminal microtubule-binding domain (C-MTBD). Here, we comprehensively analyzed the assembly structure of MTCL1 to elucidate the molecular basis of this dual activity in microtubule regulation. Our results indicate that MTCL1 forms a parallel dimer not only through multiple homo-interactions of the central coiled-coil motifs, but also the most C-terminal non-coiled-coil region immediately downstream of the C-MTBD. Among these homo-interaction regions, the first coiled-coil motif adjacent to N-MTBD is sufficient for the MTCL1 function to crosslink microtubules without affecting the dynamic property, and disruption of this motif drastically transformed MTCL1-induced microtubule assembly from tight to network-like bundles. Notably, suppression of the homo-interaction of this motif inhibited the endogenous MTCL1 function to stabilize Golgi-associated microtubules that are essential for Golgi-ribbon formation. Because the microtubule-stabilizing activity of MTCL1 is completely attributed to C-MTBD, the present study suggests possible interplay between N-MTBD and C-MTBD, in which normal crosslinking and accumulation of microtubules by N-MTBD is essential for microtubule stabilization by C-MTBD.

## Introduction

Microtubules (MTs) are polarized cylindrical polymers with a fast-growing plus end and slow-growing minus end, which play crucial roles in cellular polarity, migration, division, and vesicle transport. These diverse functions of MTs are dependent on their spatial organization and dynamic nature that are regulated by various MT-associated proteins (MAPs). In most cultured mammalian cells, the minus ends of MTs are connected to the centrosome, while their plus ends undergo a process of spontaneous growth and shrinkage, a phenomenon termed as dynamic instability. In contrast, MTs in many differentiated cells *in vivo*, including polarized epithelial cells and neurons, are not connected to the centrosome and show cell-type specific organizations [1, 2]. Recent studies have further identified a specific subset of non-centrosomal MTs, even in less differentiated cultured cells that are nucleated from the Golgi membrane, termed Golgi-derived MTs [3–5]. These non-centrosomal MTs have been shown to play important roles in establishing cell polarity that is essential for cell migration and differentiation [2].

Because non-centrosomal MTs are generally stabilized and bundled [1], it is important to clarify how cells stabilize and regulate the higher-order organization of this MT subset. However, mainly because of the intrinsic propensity of MTs to easily form bundles [6], it has been difficult to identify crosslinking MAPs in mammalian cells. For example, classical MAPs, such as tau, MAP2, and MAP4, were initially reported to crosslink MTs directly [7], because ectopic expression of their mutants strongly induced MT bundling in cultured cells [8, 9]. However, subsequent studies cast doubts on this conclusion [10, 11]. Although the resulting controversy has not been resolved completely [12, 13], it is now widely accepted that the observed MT bundling by classical MAPs reflects a secondary effect of MT stabilization induced by their direct binding [14, 15]. In fact, MT-stabilizing agents, such as taxol and a non-hydrolysable GTP analog, induce MT bundling *in vivo* [16, 17]. Conversely, removal of the acidic C-terminal regions of α- and β-tubulins by subtilisin has been demonstrated to induce MT bundling *in vitro* [18], suggesting that the reduction of electrostatic repulsive force between MTs could be another cause of their bundling. This view was supported by the fact that not only basic peptides corresponding to the MT-binding region of tau, but also unrelated basic proteins such as Yeast Lysyl-tRNA synthetase induce MT bundling *in vitro* [19]. Against this background, we can safely define a protein as an MT-crosslinking protein only when its MT-binding site does not induce MT bundling by itself, and its MT-bundling activity can be attributed to other regions [15]. Only a small number of MAPs meet these criteria for MT-crosslinking proteins, and most of them, such as PRC1/MAP65/Ase1 [20], are MAPs identified in yeast, plants, or mitotic animal cells. Thus, to clarify the molecular basis of the higher-order organization of non-centrosomal MTs observed in interphase/post-mitotic animal cells, identification and characterization of MT-crosslinking proteins in these types of cells are critically important.

**Fig 1:**
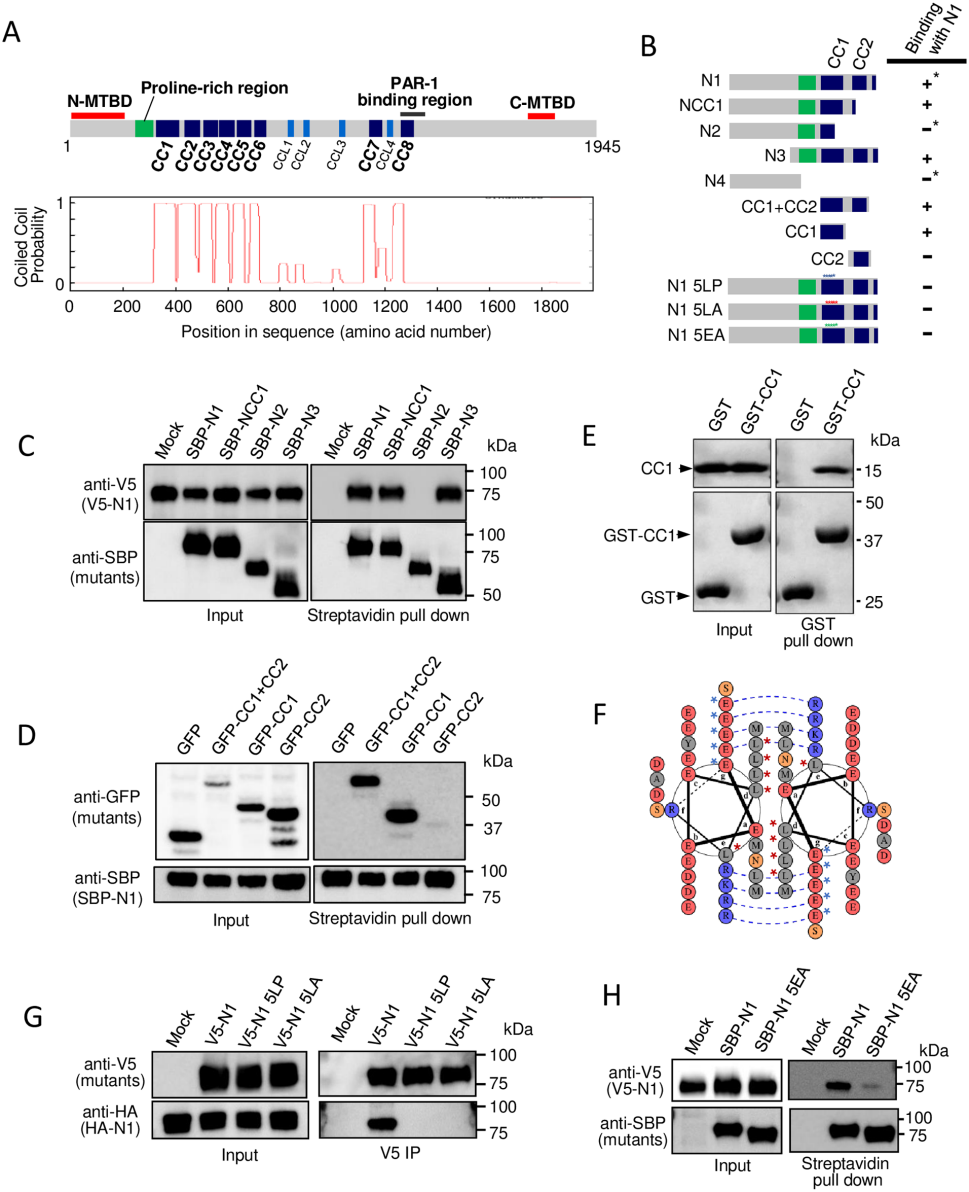
The first coiled-coil motif is critical for oligomerization of the N-MTBD of MTCL1. (A) Domain organization of mouse MTCL1 (top) and the coiled-coil probability of each motif predicted by Coils (http://www.ch.embnet.org/software/COILS_form.html) (bottom). (B) Schematic representation of the N-terminal mutants used in the following experiments and the summary of results. Asterisks indicate previously reported results [21]. (C) Streptavidin pull-down assays performed with extracts of HEK293T cells co-expressing SBP-tagged MTCL1 mutants and V5-tagged N1. (D) Streptavidin pull-down assays performed with extracts of HEK293T cells co-expressing SBP-tagged N1 and GFP-tagged mutants. (E) GST pull-down assays performed with a mixture of the purified CC1 fragment and GST or GST-CC1. (F) Helical wheel presentation of the amino acid sequence of the first half of CC1. Blue dotted lines indicate predicted salt bridges enhancing the CC1-CC1 homo-interaction. Red asterisks indicate leucine residues mutated in 5LP and 5LA mutants. Blue asterisks indicate glutamate residues mutated in the 5EA mutant. (G) Immunoprecipitation assays performed using an anti-V5 antibody and extracts of HEK293T cells co-expressing V5-tagged mutants and HA-tagged N1. (H) Streptavidin pull-down assays performed with extracts of HEK293T cells co-expressing V5-tagged N1 and SBP-tagged mutants.

Previously, we found a novel MAP named microtubule crosslinking factor 1 (MTCL1) that plays essential roles in developing the assembly structures of non-centrosomal MTs, such as epithelia-specific apicobasal MT bundles [21] and Golgi-MT networks [22]. Our recent study also revealed that MTCL1 plays indispensable roles in cerebellar Purkinje cells by supporting the stable formation of MT bundles running through the axon initial segment [23]. MTCL1 has two MT-binding domains (MTBDs) at the N- and C-terminal regions that are separated by the central region rich with coiled-coil motifs (Fig 1A). The C-terminal MTBD (C-MTBD) rich with basic residues stabilizes MTs, and thus induces MT bundling by itself when overexpressed in cultured cells [22]. Conversely, overexpressed N-terminal MTBD (N-MTBD) localizes intermittently on single MT filaments and induces MT bundling only when it is ligated to the downstream two coiled-coil motifs with an oligomerization activity [21]. This activity of N-MTBD completely meets the above criteria for MT-crosslinking proteins. These dual activities of MTCL1 to regulate MTs are highly consistent with the characteristic feature of non-centrosomal MTs [21–23]. In fact, endogenous MTCL1 localizes between MT bundles or at the intersection points of non-centrosomal MTs, and both activities have been shown to be essential for endogenous MTCL1 functions [21–23]. These results indicate that MTCL1 is a genuine MT-crosslinking protein in interphase/post-mitotic cells, which stabilizes MTs simultaneously. However, it remains to be clarified how MTCL1 integrates these regulatory roles for MTs.

In this study, we investigated the molecular basis of this MT-regulating activity of MTCL1 by comprehensively analyzing the assembly structure of the molecule. We found that MTCL1 functions as a tightly packed parallel dimer formed in a zipper-like manner through multiple homo-interacting regions distributed along the whole molecule. We also revealed the critical importance of the first coiled-coil motif for MTCL1 functions and an unexpected homo-interaction of the most C-terminal non-coiled-coil region immediately downstream of C-MTBD. Based on these data, we propose a hypothesis in which the MT-crosslinking activity mediated by N-MTBD plays essential roles in MT stabilization by C-MTBD.

## Materials and methods

### Molecular biology

The cDNA of full-length mouse MTCL1 (AK147205) was purchased from Danaform (Kanagawa, Japan). cDNA fragments corresponding to wild-type MTCL1 or its mutants were subcloned into appropriate expression vectors: pCAGGS-V5, pCAGGS-Flag- streptavidin-binding protein (SBP) [24], pEGFP (Takara Bio Inc.), pTagRFP (Evrogen JSC), pGEX (GE Healthcare), or pSRHA [25]. To establish point mutations in the N1 fragment (5LP, 5LA, and 5EA), NruI-KpnI cDNA fragments corresponding to the N-terminal region of mMTCL1 were synthesized with the appropriate mutations (Thermo Fisher Scientific) and used to replace the wild-type fragment in the V5-N1 or SBP-N1 expression vector. The amino acid numbers of mouse MTCL1 covered by the mutants are summarized in S1 Table. The C6 mutant of human MTCL1 used in previous studies [21, 22] was renamed as hCMTBD for convenience.

### Antibodies

Antibodies were purchased from commercial sources as follows: anti-GFP monoclonal antibody (mAb) (B-2), anti-SBP mAb (SB-19-C4), and anti-KIAA0802 polyclonal antibody (pAb) (W19) (Santa Cruz Biotechnology); anti-α-tubulin mAb (DM1A) and anti-acetylated tubulin mAb (Sigma-Aldrich, St. Louis, MO); anti-V5 mAb, anti-RFP pAb (Invitrogen); anti-RFP mAb (RF5R) (Thermo Fisher Scientific); anti-HA rat mAb (3F10) (Roche Applied Science Indianapolis, IN); anti-GFP pAb (MBL); anti-GM130 mAb (Cell Signaling Technology)

### Cell culture and transfection

HeLa-Kyoto (HeLa-K) and HEK293T cells were cultured in Dulbecco’s modified Eagle’s medium (Life Technologies Corporation) containing 10% fetal bovine serum, 100 U/ml penicillin, 0.1 μg/ml streptomycin, and 1 mM glutamine at 37°C in 5% CO_2_ [22]. Plasmid transfections were performed using polyethyleneimine (Polysciences, Inc.) for HEK293T cells or Lipofectamine LTX (Life Technologies Cooperation) for HeLa-K cells according to the manufacturers’ instructions.

### Pull-down and immunoprecipitation experiments

For pull-down experiments, HEK293T cells were co-transfected with an SBP-tagged MTCL1 mutant and a mutant with any other tag. Two days later, the cells were harvested and solubilized in lysis buffer (25 mM Tris-HCl, pH 7.5, 150 mM NaCl, 2.5 mM MgCl_2_, 0.1% Triton X-100, and 1 mM DTT) containing a cocktail of protease and phosphatase inhibitors (Roche Applied Science) for 30 min at 4°C, briefly sonicated, and then centrifuged at 19,000 x g for 15 min. The supernatants were incubated with streptavidin-conjugated sepharose (GE healthcare) for 2 h at 4°C to absorb the SBP-tagged mutant. The absorbed proteins were boiled in SDS sample buffer (10% β-mercaptoethanol, 125 mM Tris-HCl, pH 6.8, 2% SDS, 10% glycerol, and 0.005% bromophenol blue) for 10 min and then subjected to western blot analysis as described previously [22]. For immunoprecipitation experiments, a protein extract of HEK293T cells expressing two kinds of mutants was incubated with appropriate antibodies against one of the proteins for 2.5 h at 4°C. The immune complex was absorbed by Dynabeads protein G (Life Technologies Corporation), separated by a magnet, and then subjected to immunoblot analysis.

### Immunofluorescence microscopy

Hela-K cells were seeded at 3×10^4^/cm^2^ on coverslips coated with atelocollagen (0.5 mg/ ml; Koken), cultured overnight, and then transfected with appropriate plasmid DNA. The cells were fixed with cold methanol for 10 min at −20°C, followed by blocking with 10% (v/v) fetal bovine serum in PBS containing 0.05% Tween 20. For cold treatment experiments, the culture plates were placed on ice for 1 h before fixation. The fixed cells were subjected to standard immunostaining procedures using appropriate primary antibodies followed by respective secondary antibodies conjugated with Alexa Fluor 488, 555, or 647 (Life Technologies Corporation). The antibodies and dilutions were as follows: anti-GFP mAb (1:2,000), anti-α-tubulin mAb (1:4,000), anti-acetylated tubulin mAb (1:1,000), anti-V5 mAb (1:4,000), anti-GM130 mAb (1:2,000), and Alexa Fluor-conjugated secondary antibodies (1:2,000). Most samples were examined under an AxioImager ZI microscope (Carl Zeiss, Oberkochen, Germany) equipped with a CSU10 disc confocal system (Yokogawa Electric Corporation, Tokyo, Japan), Orca II CCD camera (Hamamatsu Photonics, Shizuoka, Japan), and 63×/1.4 NA Plan Apochromat or 100×/1.46 NA objective. Images were acquired using Meta Morph software (Molecular Devices, Sunnyvale, CA) and processed with ImageJ software to obtain appropriate brightness and contrast. To obtain wide view images for quantification, conventional fluorescence images were obtained using a 20×/0.8NA objective. Super-resolution images were acquired using a SP8-HyVolution confocal laser scanning microscope (Leica Microsystems) equipped with a 63×/1.40 oil emersion objective. Fluorescence signals were detected using HyD detectors and processed with Huygens Essential software (Scientific Volume Imaging).

### In vitro pull-down experiments

GST-tagged CC1 and C9 were purified from the soluble fraction of an *E*.*coli* extract using glutathione sepharose (GE healthcare) by standard methods. To obtain tag-free fragments, the GST-fusion proteins bound to the glutathione resin were subjected to cleavage using precision protease (GE healthcare). After dialysis against PBS for 24 h, GST-CC1 or GST-C9 were mixed with CC1 or C9 fragments, respectively, at a final concentration of 0.5 mg/ml and then incubated at 4°C for 3 h. GST-tagged proteins were recovered by incubation with glutathione sepharose and analyzed by SDS-polyacrylamide gel electrophoresis (PAGE) followed by Coomassie Brilliant Blue (CBB) staining.

### Purification of full-length MTCL1 from HEK293T cells

To purify full-length mouse MTCL1 from HEK293T cells, the nucleotides of the mouse cDNA were codon-optimized for human cells (Thermo Fisher Scientific). HEK293T cells were seeded at 1.2×10^7^/15-cm dish and transiently cotransfected with an expression vector for humanized Flag-SBP-MTCL1 and the pAdVAntage^™^ Vector. After incubation at 37°C for approximately 48 h, the cells were lysed in F-buffer (20 mM Tris-HCl, pH 7.5, 150 mM NaCl, 0.25 M sucrose, 1% Triton X-100, and 0.5% NP-40) containing the cocktail of protease and phosphatase inhibitors for 30 min at 4°C, briefly sonicated, and then centrifuged at 15,000 x *g* for 30 min. Then, the supernatant was incubated with streptavidin-conjugated magnetic beads (GE Healthcare) that had been prewashed with antibody dilution buffer containing bovine serum albumin (BSA) to prevent nonspecific binding of the beads to other cellular proteins. After incubation for a suitable time period, the beads were separated by a magnet and washed four times with wash buffer consisting of a similar composition as the F-buffer. Finally, the protein was eluted with 10 mM biotin in PBS.

### Microtubule spindown for visual analysis

To observe the assembly structure of MTs in the presence or absence of purified MTCL1, taxol-stabilized MTs (2.5 μM relative to the tubulin heterodimer) were mixed with purified MTCL1 (0.4 or 1.2 μM) at 35°C for 30 min and then fixed at room temperature for 3 min by adding 10 volumes of 1% glutaraldehyde in BRB80 buffer (80 mM PIPESKOH, pH 6.8, 1 mM MgCl_2_, and 1 mM EGTA). After diluting the mixture with 25 volumes of BRB80 buffer, 120 μl of the MT solutions were sedimented onto coverslips, postfixed with cold methanol, blocked with BSA, and processed for immunofluorescence staining using anti-tubulin and anti-MTCL1 antibodies. Super-resolution images were acquired using a SP8-HyVolution confocal laser scanning microscope (Leica Microsystems) equipped with a 100×/1.40 oil emersion objective. Fluorescence signals were detected using HyD detectors and processed with Huygens Essential software (Scientific Volume Imaging).

## Results

### The first coiled-coil motif is critical for oligomerization of the N-MTBD of MTCL1

Previously, we demonstrated that N-MTBD acquires a homo-interaction activity only when it is connected to the downstream two coiled-coil motifs, CC1 and CC2, in a fragment termed N1 (Fig 1B, see asterisks) [21]. Here, to further confine the responsive region for the homo-interaction, we performed additional deletion mapping, and finally found that CC1, but not CC2, is required and sufficient to interact with N1 (Fig 1B–1D). Because CC1 shows a homo-interaction activity *in vitro* and *in vivo* (Fig 1E and S1 Fig), these results indicate that the CC1-CC1 interaction mediates homo-oligomerization of the N-terminal region of MTCL1. To confirm this conclusion, we mutated leucine residues appearing every seven amino acids in the first half of CC1 to proline (5LP) in the N1 fragment, which disrupts the α-helix formation in CC1 (Fig 1F, red asterisks, S2A and S2B Fig). Fig 1G shows that the N1, but not N1 5LP, mutant interacted with N1, indicating the critical importance of the α-helical structure in CC1. Even when the leucine residues were mutated to alanine to preserve the α-helical structure of CC1 (S2B Fig), the N1 mutant (5LA) lost its ability to interact with wild-type N1 (Fig 1G). This result indicates that not only α-helix formation but also the coiled-coil interaction mediated by the regularly packed leucine residues located on the same side of the α-helix is crucial for the homo-interaction of CC1.

The coiled-coil motif has a characteristic seven residue repeat, (***a-b-c-d-e-f-g***)_n_, with hydrophobic residues at positions ***a*** and ***d***, and polar residues generally elsewhere [26]. Accumulating evidence has demonstrated that the presence of hydrophobic residues at the interacting surface, *per se*, is not always sufficient for stable and specific coiled-coil assembly, and interhelical salt bridges can provide additional stability to the coiled-coil oligomers [27]. In fact, the helical wheel presentation of the first half of CC1 predicted the consecutive appearance of acidic residues (glutamate) at the ‘***g***’ position and basic residues (lysine or arginine) at the ‘***e***’ position along the α-helical structure (Fig 1F, blue asterisks, S2A Fig). These residues could undergo ionic interactions flanking the core hydrophobic interaction interface that is essential for the CC1-CC1 homo-interaction. This idea was supported by results indicating that substitution of the five glutamate residues successively observed at the ‘***g***’ position in CC1 (Fig 1F, blue asterisks) to alanine (5EA) disrupted the homo-interaction of the N1 fragment (Fig 1H). Collectively, these results suggest that the coiled-coil interaction of CC1 together with salt bridges mediate the homo-interaction of the N-terminal region of MTCL1.

### The coiled-coil interaction of CC1 plays an essential role in bundling MTs without stabilization

**Fig 2:**
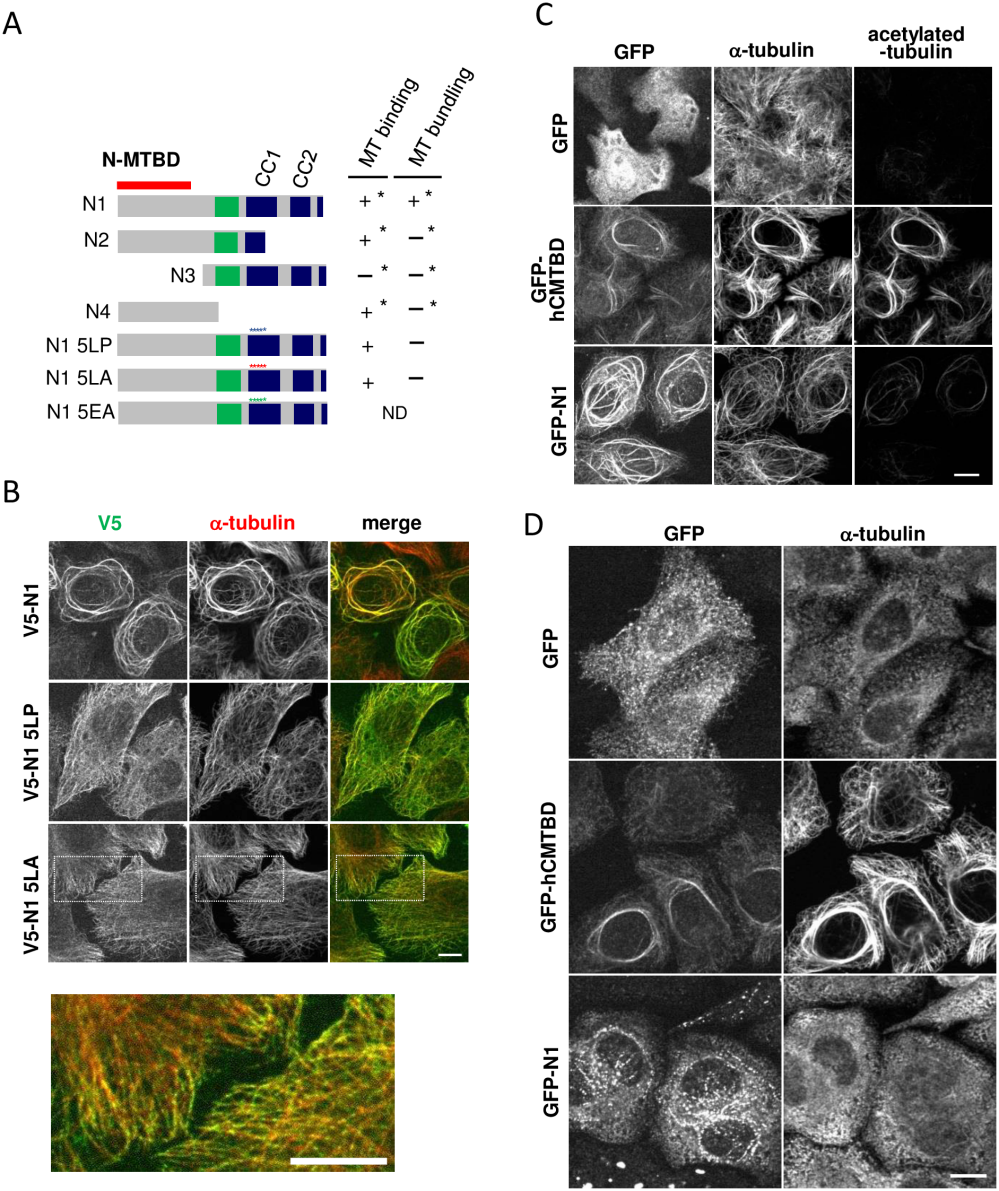
The coiled-coil interaction of CC1 plays an essential role in N1-mediated crosslinking of dynamic MTs. (A) Schematic representation of the N-terminal mutants used in the following experiments and the summary of results. Asterisks indicate previously reported results [21]. ND indicates not determined. (B) Immunostaining of V5 and α-tubulin in HeLa-K cells expressing V5-N1 wild-type, 5LP, or 5LA. The bottom panel is an enlarged view of the rectangle region of V5-N1 5LA-expressing cells. Scale bars, 10 μm. (C) Immunostaining of GFP, α-tubulin, and acetylated tubulin in HeLa-K cells expressing GFP, GFP-hCMTBD, and GFP-N1. Scale bar, 10 μm. (D) Immunostaining of GFP and α-tubulin in HeLa-K cells expressing GFP, GFP-hCMTBD, and GFP-N1 after cold treatment on ice for 1 h. Scale bar, 10 μm.

A previous study has demonstrated that ectopic expression of N1 strongly induces MT bundling in HeLa-K cells, whereas N-terminal fragments lacking coiled-coil motifs (N2 and N4) intermittently associate with the MT lattice but not bundle MTs (Fig 2A, asterisks) [21]. Here, we extended these results by revealing that the N1 fragment with 5LA or 5LP mutations in CC1 lost the MT-crosslinking activity but retained the ability to bind individual MT filaments similarly to N2 and N4 (Fig 2B). These data strongly support our notion that N1-induced MT bundling is not due to MT stabilization, but represents genuine MT crosslinking in which MTs are bundled together by the coiled-coil interaction-mediated multimerization of N-MTBD.

This conclusion was reinforced by the following experiments. First, N1-induced MT bundles did not exhibit strong immunostaining for acetylated tubulin, a marker of stabilized MTs (Fig 2C) [28]. This result is in sharp contrast to MT bundles induced by C-MTBD [21, 22], which significantly enhanced the acetylated-tubulin signal (Fig 2C). Second, MT filaments within N1-induced MT bundles showed severe depolymerization after cold treatment, whereas MT bundles associated with C-MTBD exhibited strong resistance (Fig 2D). These results indicate that the N-terminal region of MTCL1 crosslinks MTs without causing stabilization. This feature of MT crosslinking was not changed even when N-MTBD works within the full-length molecule, because deletion of C-MTBD was sufficient to abolish the MT stabilization activity of full-length MTCL1 (S3 Fig). This finding indicates that the MT stabilization activity is completely attributed to C-MTBD.

### MTCL1 forms a parallel dimer thorough multiple homo-interactions along the molecule

Computational analysis of the amino acid sequence of MTCL1 indicates a significantly high probability of dimer, but not trimer, formation [21]. In fact, as previously demonstrated for coiled-coil peptides with interhelical hydrogen bonding [29, 30], the purified CC1 fragment exhibited a band with the molecular mass of a dimer in SDS-PAGE analysis without heat pretreatment (S4A Fig). Dimer formation of CC1 was further supported by crosslinking experiments using disuccinimidyl tartrate (DST), which predominantly produced a band with the molecular mass corresponding to a dimer (S4B Fig).

Considering that, in general, CC motifs can form antiparallel dimers, and not all the CC motifs of MTCL1 might show homo/hetero-interactions, the above detection of the CC1-CC1 homo-interaction still formally allows various modes of MTCL1 assembly, some examples of which are shown in S4C Fig. Thus, to gain an insight into the assembly structure of full-length MTCL1, we identified all regions involved in the homo-interaction of MTCL1 by systematically dividing the MTCL1 molecule and subjecting the fragments to pull-down assays.

**Fig 3:**
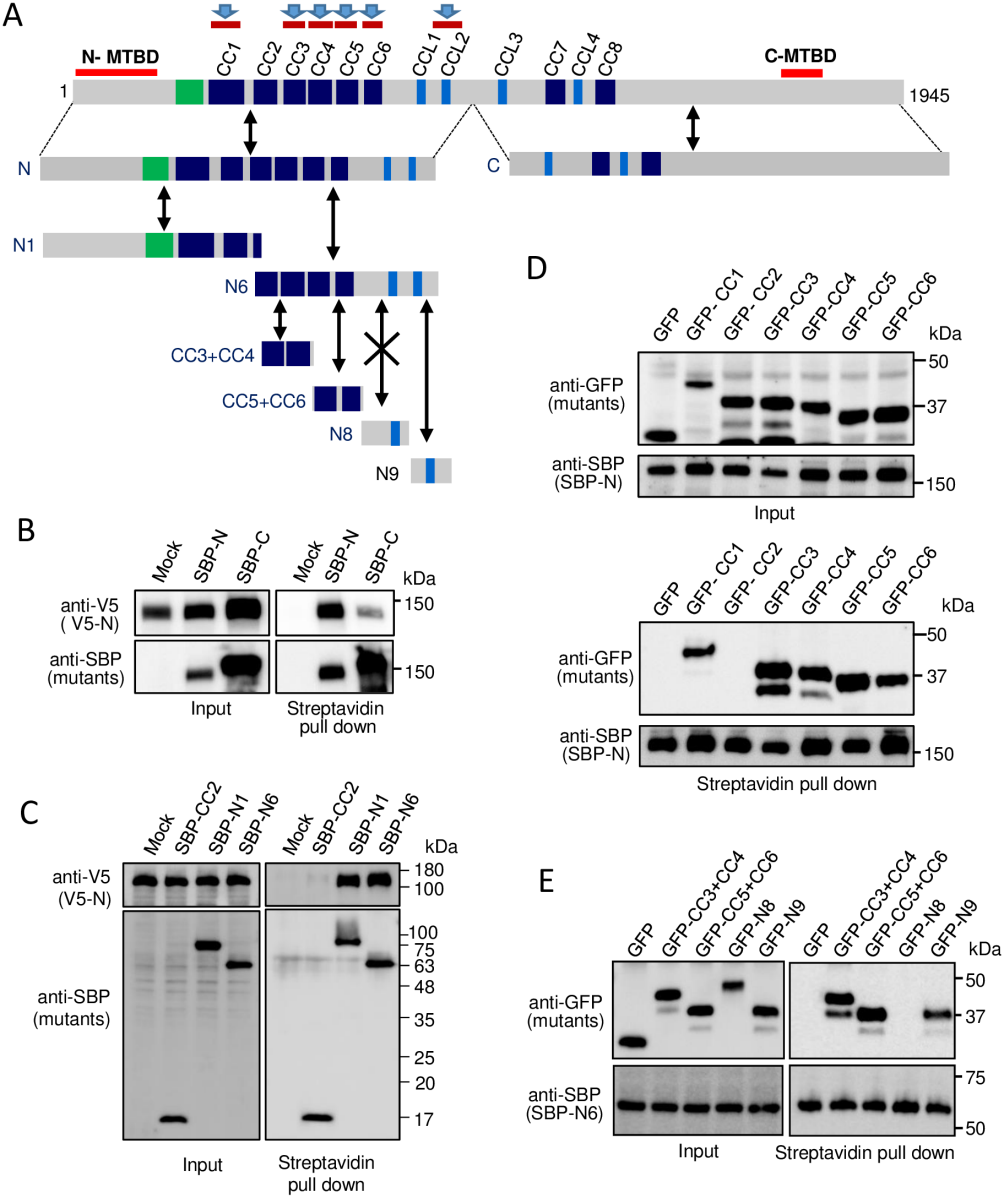
Identification of homo-interaction regions in the N-terminal half of MTCL1. (A) Schematic representation of the MTCL1 mutants used in the following experiments and the summary of results. Bidirectional black arrows indicate binding, and that with a cross mark indicates no binding. Brown bars with a blue arrow (top) indicate the identified homo-interaction regions. (B) Streptavidin pull-down assays performed with extracts of HEK293T cells co-expressing SBP-N or C and V5-tagged N. (C) Streptavidin pull-down assays performed with extracts of HEK293T cells co-expressing SBP-tagged CC2, N1 or N6 and V5-tagged N. CC2 was included to verify the specificity of the interactions. (D) Streptavidin pull-down assays performed with extracts of HEK293T cells co-expressing SBP-tagged N and individual N-terminal CCs with a GFP tag. (E) Streptavidin pull-down assays performed with extracts of HEK293T cells co-expressing SBP-tagged N6 and GFP-tagged CC3+CC4, CC5+CC6, N8, or N9.

First, we divided the MTCL1 molecule into two halves and found that not only the N-terminal fragment (N), but also the C-terminal fragment (C) individually showed a homo-interaction without cross-interaction (Fig 3B and S5A Fig). When N was further divided into two halves (N1 and N6), not only N1, but also N6 showed the interaction with the N fragment, suggesting that coiled-coil motifs other than CC1 are also involved in the homo-interaction of MTCL1 (Fig 3C). Consistently, all N-terminal CCs except CC2 showed an interaction with the N fragment (Fig 3D), indicating that the lack of the homo-interacting activity is a specific feature of CC2. N1 and N6 did not show a cross-interaction (S5B Fig), supporting the notion that N-terminal CCs other than CC2 show a homo-interaction, but not a hetero-interaction. In fact, homo-interactions were observed for all N-terminal CCs except CC2 (S5C–S5F Fig). We also confirmed that CC1, CC5 and CC6 do not show hetero-interactions with any other N-terminal CCs (S6 Fig). Subdividing the N6 fragment further revealed that, in addition to typical CCs, the fragment containing CCL2 (N9), but not CCL1 (N8), interacted with N6 probably through a homo-interaction (Fig 3E). The above results are summarized in Fig 3A.

**Fig 4:**
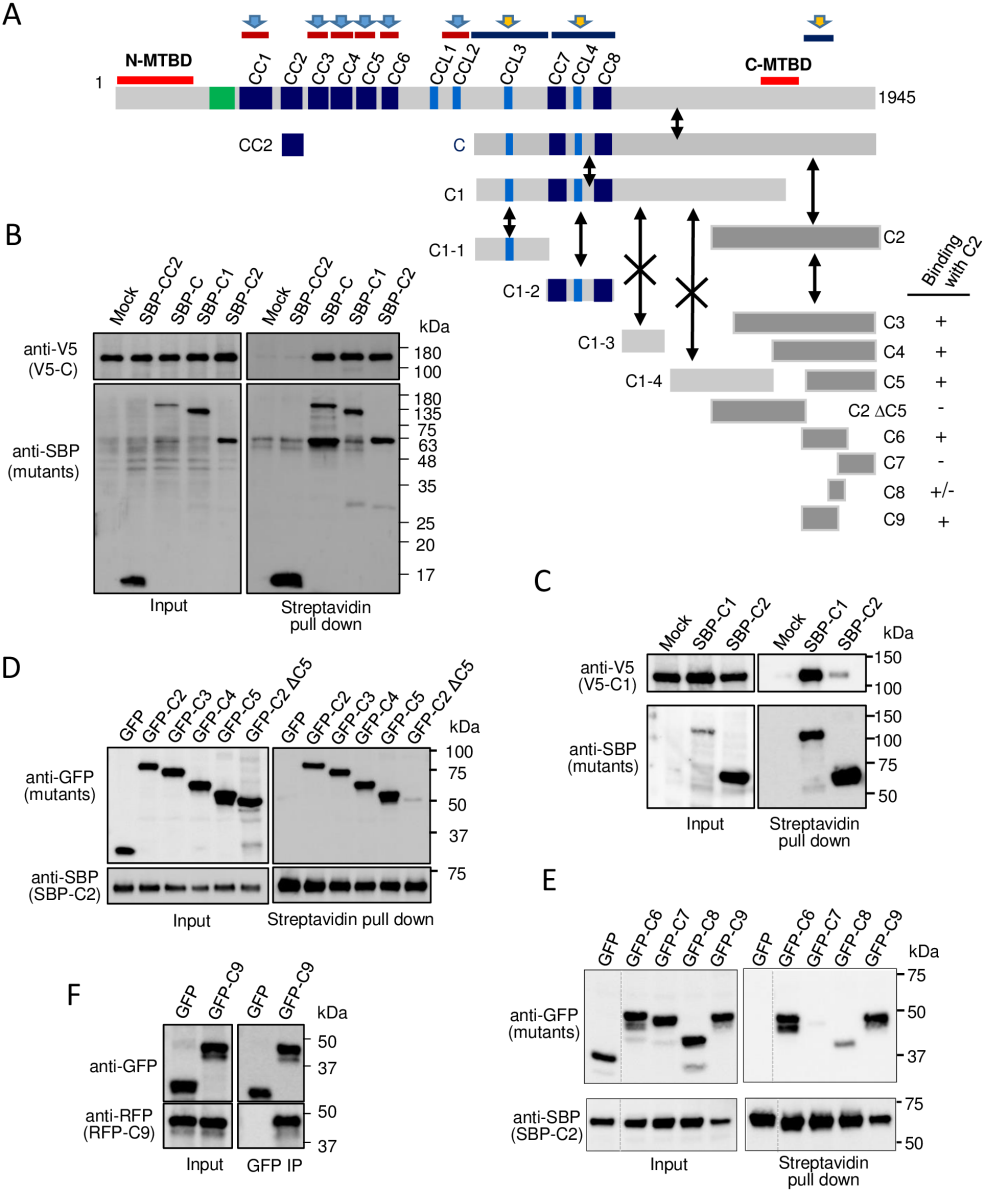
Identification of homo-interaction regions in the C-terminal half of MTCL1. (A) Schematic representation of the MTCL1 mutants used in the following experiments and the summary of results. Bidirectional black arrows indicate binding, and those with a cross mark indicate no binding. Deep-blue bars with a yellow arrow (top) indicate the identified homo-interaction regions. (B) Streptavidin pull-down assays performed with extracts of HEK293T cells co-expressing SBP-CC2, C, C1 or C2 and V5-tagged C. CC2 was included to verify the specificity of the interactions. (C) Streptavidin pull-down assays performed with extracts of HEK293T cells co-expressing SBP-C1 or C2 and V5-tagged C1. (D) Streptavidin pull-down assays performed with extracts of HEK293T cells co-expressing GFP-C2, C3, C4, C5 or C2ΔC5 and SBP-tagged C2. (E) Streptavidin pull-down assays performed with extracts of HEK293T cells co-expressing SBP-C2 and GFP-tagged C6, C7, C8, or C9. (F) Immunoprecipitation assays performed using an anti-GFP antibody and extracts of HEK293T cells co-expressing GFP or GFP-C9 and RFP-C9.

Division of the C-terminal half of the molecule (C) revealed that not only the first two-thirds of fragment rich in coiled-coil motifs (C1), but also the most C-terminal fragment without the coiled-coil motif (C2) showed an interaction with C without a cross-interaction (Fig 4B and 4C). The homo-interaction of C1 was mediated by regions containing CCL3 (C1-1), or CC7, CCL4, and CC8 (C1–2), but not regions without coiled-coil motifs (C1–3 and C1–4) (S7A Fig). Conversely, the homo-interaction of the most C-terminal region was mediated by a very restricted region (C9) containing 73 amino acids located immediately downstream of the C-MTBD (Fig 4D–4F and S7B Fig). Because purified GST-C9 did not pull down the C9 fragment *in vitro* (S7C Fig), the homo-interaction between C9 may be indirect and probably mediated by an unknown binding partner(s).

In summary, the homo-interaction of MTCL1 is mediated not only by the central coiled-coil rich region, but also the C-terminal short segment just downstream of C-MTBD (Fig 4A top). These results exclude the possibility of antiparallel dimerization of MTCL1 (S4C Fig) and indicate that MTCL1 forms a tightly packed parallel dimer by interacting each other in a zipper-like manner through multiple points distributed along the whole molecule.

### MTCL1 crosslinks MTs *in vitro*

**Fig 5:**
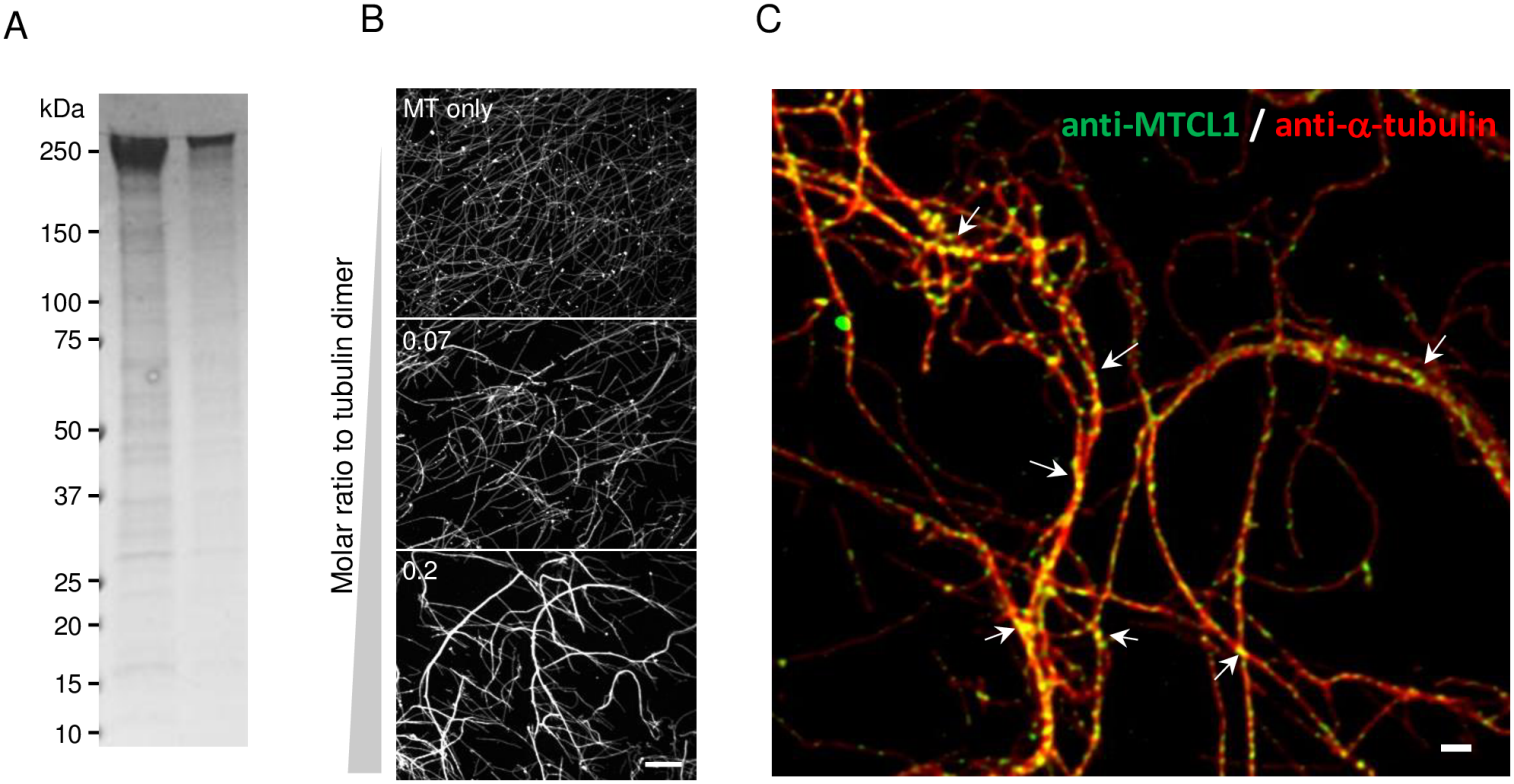
MTCL1 crosslinks MTs *in vitro*. (A) CBB staining of purified SBP-MTCL1 (lane 1; 2 μg, lane 2; 0.4 μg) separated by SDS-PAGE. (B) Visualization of taxol-stabilized MTs mixed with purified SBP-MTCL1 using an anti-tubulin antibody. Molar ratios of SBP-MTCL1 to the tubulin dimer (0, 0.07 or 0.2) are indicated. (C) Super-resolution image of MTs mixed with SBP-MTCL1 (molar ratio to tubulin dimer = 0.2) and double stained with anti-α-tubulin and anti-MTCL1 antibodies.

To directly analyze the assembly structure of the whole MTCL1 molecule, we tried to purify MTCL1 as an SBP-tagged protein from HEK293T cells (Fig 5A). Unfortunately, the purity and yields of the resultant samples were insufficient for subsequent biophysical or electron microscopic analyses to conclusively determine the assembly structure of MTCL1. However, the purified protein was used to demonstrate that MTCL1 directly induces MT bundles *in vitro* in a dose-dependent manner (Fig 5B). MTCL1 immunostaining and subsequent analysis by super-resolution microscopy revealed that MTCL1 bound to the lattice of MT bundles in patches, and frequently concentrated at crossing or divergent points of MTs in bundles (arrows in Fig 5C). These results support the notion that MTCL1 is a genuine MT-crosslinking protein that directly interlinks MTs into bundles and networks. These localizations of purified MTCL1 on MTs are highly consistent with those observed for endogenous MTCL1 [21–23], but different from those of ectopically expressed MTCL1 that heavily coated MT bundles without any gap (S3 Fig) [21, 22]. Therefore, the above localization of purified MTCL1 also indicated that strong activity of overexpressed MTCL1 inducing tight and thick MT bundles does not correctly reflect the physiological function of MTCL1 that should function at rather limited concentrations compared with the overexpressed condition (see Discussion).

### The coiled-coil interaction through CC1 is essential for tight crosslinking of MTs by MTCL1

**Fig 6:**
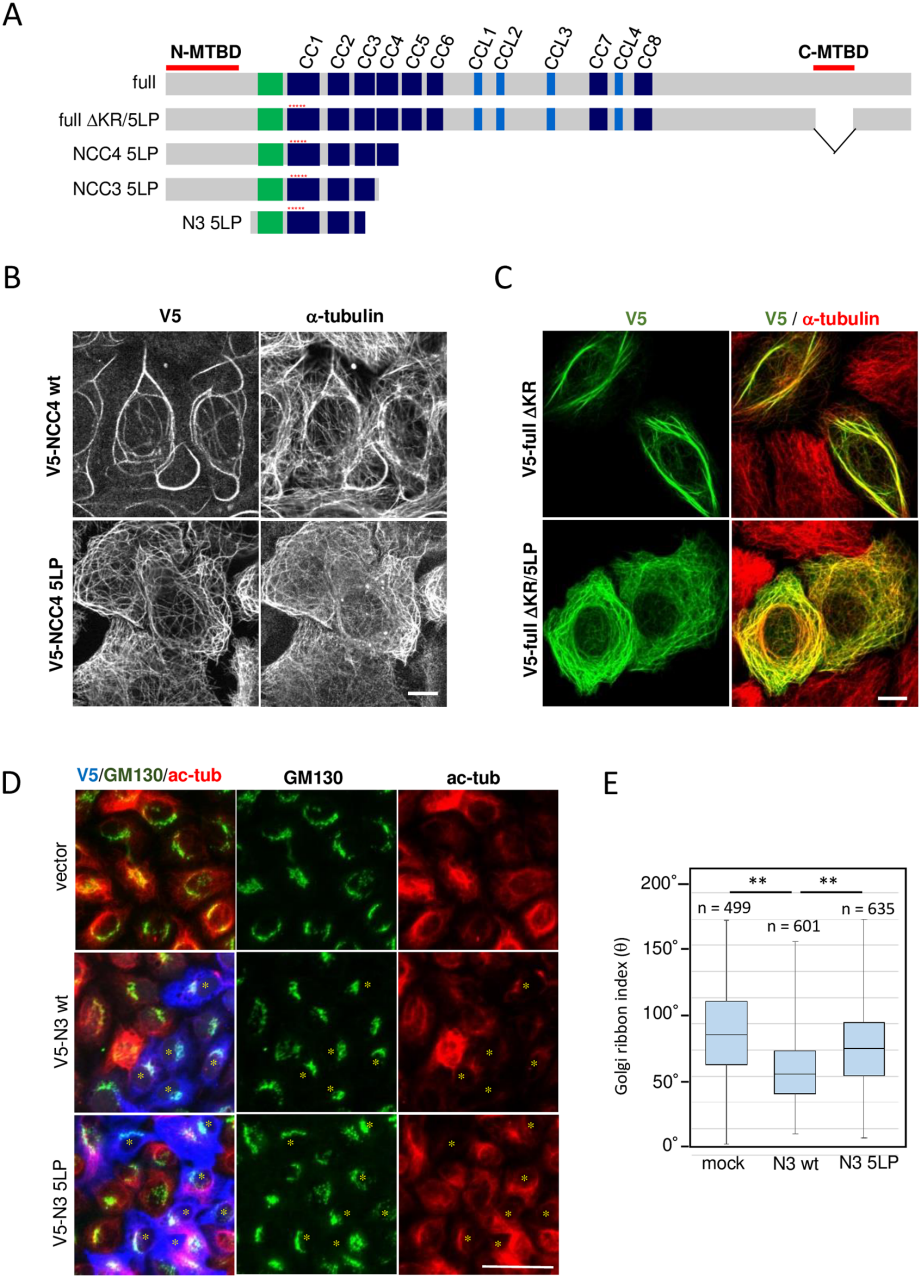
The coiled-coil interaction through CC1 is critically important for tight crosslinking of MTs. (A) Schematic representation of the MTCL1 mutants used in the following experiments. Red asterisks indicate the position of the 5LP mutation. (B) Immunostaining of V5 and α-tubulin in HeLa-K cells expressing V5-NCC4 wt or its 5LP mutant. Scale bar, 10 μm. (C) Immunostaining of V5 and α-tubulin in HeLa-K cells expressing V5-full ΔKR or its 5LP mutant. Scale bar, 10 μm. (D) Immunostaining of V5 (blue), GM130 (cis-Golgi marker) (green), and acetylated tubulin (red) in HeLa-K cells expressing V5-N3 wt or its 5LP mutant. Asterisks indicate cells expressing ectopic proteins. Scale bar, 50 mm. (E) Box plot presentation of the Golgi-ribbon index corresponding to the laterally expanding angle of the Golgi apparatus around nuclei (θ). The lines within each box represent medians. Data represent the results of the indicated number (n) of cells in three independent experiments. **P < 0.01, estimated by the two-tailed Mann-Whitney U-test.

The present results highlight the uniqueness of CC2 that does not show a homo-interaction despite the high score of coiled-coil formation (Fig 1A). Although the reason for this incompetence of CC2 is unclear, this specific feature of CC2 indicated a possibly important role in the preceding CC, CC1, for MTCL1 assembly. Thus, we examined the effect of disrupting the CC1-CC1 interaction on the MT-crosslinking activity of MTCL1 mutants longer than N1. Consistent with the results showing that CC3 and CC4 exhibit a homo-interaction (S5C and S5D Fig), N-terminal fragments of MTCL1 covering N-MTBD to CC3 (NCC3) or CC4 (NCC4) induced MT bundling in HeLa-K cells, even when the CC1-CC1 homo-interaction was disrupted by the 5LP mutation (Fig 6A and 6B, S8 Fig). However, the 5LP mutation significantly affected the MT assembly structure induced by these fragments. For example, in contrast to NCC4 wild-type that induced tight and thick MT bundles, N-CC4 5LP induced faint MT bundles that tended to form networks covering the whole cytoplasm (Fig 6B). Interestingly, similar results were also obtained when the 5LP mutation was introduced into the MTCL1 full-length mutant, full ΔKR [21] (Fig 6C). In this mutant, C-MTBD was disrupted to remove its strong MT-bundling activity indirectly induced by MT stabilization (Fig 2C). These results suggest that the homo-interaction via CC1 plays a unique role in MT crosslinking by MTCL1 even in the full-length molecule. Considering that CC1 is located at the immediate downstream region of N-MTBD, the above results suggest that CC1 may facilitate close crosslinking of MTs by squeezing the neck of the N-terminal region of MTCL1.

Finally, we examined the physiological significance of the CC1-mediated coiled-coil interaction by overexpressing V5-tagged N3 in HeLa-K cells, which would interfere with the CC1 interaction of endogenous MTCL1 (Fig 6D and 6E). A previous study has demonstrated that MTCL1 plays essential roles in the development of Golgi-ribbon structures by stabilizing and crosslinking Golgi-derived MTs in HeLa-K cells [22]. Notably, V5-N3-expressing cells exhibited a reduction in the accumulation of acetylated MTs as well as lateral expansion of the Golgi complex into the ribbon-like structure (Fig 6D and 6E) similarly to MTCL1 knockdown cells [22]. Overexpression of V5-N3 with the 5LP mutation induced significantly weaker effects, suggesting that the observed deleterious effects of N3 on MTCL1 functions are due to the dominant negative effect on the CC1 homo-interaction. These results indicate that the precise assembly structure of MTCL1 is critically important for the endogenous functions of MTCL1.

## Discussion

Several actin-binding proteins are known to dynamically regulate higher-order structures of actin filaments by crosslinking or bundling them with various spaces and angles [31]. In contrast, molecules responsible for the higher-order structures of MTs have not been well studied, mainly because the characteristic properties of MTs are different from those of actin filaments. In this study, we finally established that MTCL1 is the genuine MT-crosslinking protein that gathers MTs via its N-terminal region without significantly affecting their dynamic property. Furthermore, we revealed that MTCL1 forms a parallel dimer in which the molecules are tightly packed through multiple points distributed along the whole molecule. This architecture of the MTCL1 dimer implies possible coupling of the MT-crosslinking and stabilization activities of MTCL1 (see below).

The present results unexpectedly demonstrated that, among eight motifs of the typical coiled-coil structure, only CC2 cannot exhibit an interaction with any other CC including itself. The structural basis of this unique feature of CC2 is still unknown. However, the result highlights the distinctive role of the preceding CC, CC1, whose homo-interaction is enhanced by salt bridges (Fig 1H), shows resistance to SDS denaturation (S4A Fig), and has sufficient activity to crosslink MTs by itself (Fig 2B). Interestingly, disruption of the CC1 homo-interaction is sufficient to profoundly affect MTCL1-induced assembly structures of MTs (Fig 6B and 6C): MTCL1 lacking the CC1-CC1 interaction induced network-like MT assembly instead of tight bundles as if the presence of the CC1-CC1 interaction determines the angle at which MTCL1 crosslinks MTs. This may correspond to the predicted flexible feature of the N-MTBD that not only contains many prolines in its own sequence, but is also linked to CC1 via a proline-rich sequence (S2A Fig). Here, we observed that inhibition of the CC1-CC1 interaction significantly affected the physiological functions of MTCL1 (Fig 6D). Taken together, these results raise the intriguing possibility that regulation of the CC1 homo-interaction is one of the mechanisms to control MTCL1 functions and MT assembly structures endogenously. Although any phosphorylation site has not been identified in CC1, regulated binding of some proteins to CC1 or the preceding proline-rich region might affect the CC1-CC1 interaction by steric hindrances.

In addition to the MT-crosslinking activity via N-MTBD, the MT-stabilizing activity mediated by C-MTBD has been shown to be indispensable for endogenous MTCL1 functions to regulate non-centrosomal MT organization [22, 23]. In this study, we finally confirmed that this activity is completely attributed to C-MTBD (S3 Fig), and MT crosslinking by MTCL1 does not significantly contribute to MT stabilization (Fig 2D). In addition, we found that C-MTBD is flanked by the homo-interaction regions of MTCL1 located at the central region and the immediate downstream of C-MTBD (Fig 4A). This observation raises an intriguing possibility that, in contrast to N-MTBD, C-MTBD is sterically tucked away within the MTCL1 dimer and inhibited to interact with or stabilize MTs freely unless the extended MTCL1 dimers are closely located along the MTs crosslinked by N-MTBD. This is consistent with the results indicating that, in contrast to ectopically overexpressed MTCL1 [22] or C-MTBD (Fig 2C), not all signals of endogenous MTCL1 were located on stabilized MTs, and some are detected on the lateral side of single MT filaments that are not stabilized (S9 Fig, arrowheads) [22]. MT stabilization is mainly observed in MT bundles on which endogenous MTCL1 are highly accumulated (S9 Fig) [22]. These results may indicate that MT stabilization by C-MTBD efficiently occurs only when MTCL1 associates with MTs at a high concentration, and thereby crosslinks and accumulates many MTs through the N-MTBD activity. Interestingly, we observed that suppression of the CC1 homo-interaction resulted in similar phenotypes to MTCL1 knockdown, including reduced accumulation of acetylated tubulin around the Golgi apparatus and impaired Golgi-ribbon development (Fig 6D) [22]. Because MT stabilization by CMTB is essential for the MTCL1 function to promote Golgi-ribbon formation [22], these results are consistent with the above idea that crosslinking of MTs via N-MTBD is tightly coupled with the MT stabilization activity of C-MTBD. An increased crossing angle of MT assembly might impair efficient stabilization of MTs by C-MTBD. Unfortunately, strong MT stabilization activity of ectopically-expressed MTCL1 have prevented us to examine these hypotheses in the present study. Future identification of appropriate conditions to suppress MTCL1 expression levels will enable us to further investigate the coordinated regulation of crosslinking and stabilization of MTs by MTCL1 implicated in this study.

## Supporting information

S1 Fig CC1 shows a homo-interaction *in vivo*. Immunoprecipitation assays were performed using an anti-GFP antibody and extracts of HEK293T cells co-expressing GFP or GFP-tagged CC1 and RFP-tagged CC1.
(PDF)

S2 Fig The amino acid sequence of the MTCL1 N-terminal region and the predicted secondary structure of the first half of CC1. (A) The amino acid sequence of mouse MTCL1 from 1 to 450 is shown. Red and green boxes indicate N-MTBD and the proline-rich region, respectively. Proline residues are red. The sequences corresponding to CC1 and CC2 are boxed by black, in which residues in the “*d*” position of the heptad repeat (***a-b-c-d-e-f-g***)_**n**_ are blue. The amino acids in the first half of CC1 are bold, and the mutated residues are indicated by asterisks. Note that the heptad pattern of the first and latter half of CC1 is interrupted by a single residue, glutamate (red arrow). (B) Secondary structures of the first half of CC1 wt and its mutants were predicted by the PSIPRED program (http://bioinf.cs.ucl.ac.uk/psipred/). Positions of mutated leucine residues are indicated by red rectangles. Seven residue repeats, (***a-b-c-d-e-f-g***), are indicated by black brackets under the sequences.
(PDF)

S3 Fig MTCL1 cannot stabilize MTs without C-MTBD. Immunostaining of V5 and α-tubulin in HeLa-K cells expressing V5-MTCL1 full wt and its mutant lacking C-MTBD (fullΔKR). Right panels show cells fixed after cold treatment on ice for 1 h. Scale bars, 10 μm.
(PDF)

S4 Fig CC1 forms a dimer. (A) The CC1 fragment purified from *E*.*coli* (with a calculated molecular mass of ~10.5 kDa) was solubilized in SDS sample buffer containing 2% SDS with or without heat treatment (100°C, 10 min) and then subjected to SDS-PAGE analysis and CBB staining. (B) Crosslinking result of monomeric CC1 with the chemical crosslinking reagent DST. The purified CC1 fragment (0.52 mg/ml) dissolved in PBS was incubated for 30 min at 25°C in the presence of various concentrations of DST (lanes 2–4: 0.1, 0.3 and 0.9 mg/ml, respectively). The reaction was stopped by addition of a Tris-HCl stock solution (pH 7.5) at a final concentration of 50 mM followed by incubation for 15 min at 25°C.(C) Possible modes of MTCL1 assembly based on the CC1-CC1 homo-interaction.
(PDF)

S5 Fig Supplementary data for identification of homo-interaction regions in the MTCL1 N-terminus. Streptavidin pull-down assays performed with extracts of HEK293T cells co-expressing the indicated MTCL1 mutants. (A) The C fragment interacts with itself, but not with the N fragment. (B) The N1 fragment interacts with itself, but not with the N6 fragment. (C–F) Immunoprecipitation assays by anti-GFP antibody examining interactions of RFP-CC3 (C), CC4 (D), CC5 (E) or CC6 (F) with the indicated GFP proteins.
(PDF)

S6 Fig N-terminal CCs show highly specific homo-interaction. Streptavidin pull-down assays performed with extracts of HEK293T cells co-expressing SBP-CC1 (A), CC5 (B) or CC6 (C) and the indicated GFP-fusion proteins of the N-terminal CCs.
(PDF)

S7 Fig Supplementary data for identification of homo-interaction regions in the MTCL1 C-terminus. (A) Streptavidin pull-down assays performed with extracts of HEK293T cells co-expressing SBP-tagged mutants and V5-tagged C1. (B) Alignment of the amino acid sequence of C9. (C) GST pull-down assays performed with a mixture of purified C9 fragment and GST or GST-C9.
(PDF)

S8 Fig Effect of the 5LP mutation in CC1 on the MT-crosslinking activity of V5-NCC3. Immunostaining of V5 and α-tubulin in HeLa-K cells expressing V5-NCC3 wt and its 5LP mutant. Scale bars, 10 μm.
(PDF)

S9 Fig MTs decorated with MTCL1 do not necessarily correspond to stabilized MTs. Hela-K cells were triply immunostained with antibodies against MTCL1, α-tubulin and acetylated tubulin, and analyzed by super-resolution microscopy. The bottom panels correspond to enlarged views of the rectangle region indicated in the top panels. Arrowheads indicate MTCL1 signals on MTs which are not strongly stained with anti-acetylated tubulin antibody. Scale bars, 5 μm (top panels) or 2 μm (bottom panels).
(PDF)

S1 Table Amino acid numbers of mouse MTCL1 covered by each mutant. (PDF)

## References

[1] Bartolini F, Gundersen GG. Generation of noncentrosomal microtubule arrays. Journal of cell science. 2006;119(Pt 20):4155–63. Epub 2006/10/14. doi: 10.1242/jcs.03227 . 17038542 10.1242/jcs.03227

[2] Nishita M, Satake T, Minami Y, Suzuki A. Regulatory mechanisms and cellular functions of non-centrosomal microtubules. J Biochem. in press. 10.1093/jb/mvx018 28338985

[3] Efimov A, Kharitonov A, Efimova N, Loncarek J, Miller PM, Andreyeva N, et al Asymmetric CLASP-dependent nucleation of noncentrosomal microtubules at the trans-Golgi network. Developmental cell. 2007;12(6):917–30. Epub 2007/06/05. doi: 10.1016/j.devcel.2007.04.002 ; 17543864 10.1016/j.devcel.2007.04.002 PMC2705290

[4] Rivero S, Cardenas J, Bornens M, Rios RM. Microtubule nucleation at the cis-side of the Golgi apparatus requires AKAP450 and GM130. The EMBO journal. 2009;28(8):1016–28. Epub 2009/02/27. doi: 10.1038/emboj.2009.47 ; 19242490 10.1038/emboj.2009.47 PMC2683699

[5] Sanders AA, Kaverina I. Nucleation and Dynamics of Golgi-derived Microtubules. Frontiers in neuroscience. 2015;9:431 Epub 2015/12/01. doi: 10.3389/fnins.2015.00431 ; 26617483 10.3389/fnins.2015.00431 PMC4639703

[6] Safinya CR, Chung PJ, Song C, Li Y, Ewert KK, Choi MC. The effect of multivalent cations and Tau on paclitaxel-stabilized microtubule assembly, disassembly, and structure. Advances in colloid and interface science. 2016;232:9–16. Epub 2015/12/20. doi: 10.1016/j.cis.2015.11.002 ; 26684364 10.1016/j.cis.2015.11.002 PMC4864139

[7] Lewis SA, Cowan N. Microtubule bundling. Nature. 1990;345(6277):674 Epub 1990/06/21. doi: 10.1038/345674a0 . 2113613 10.1038/345674a0

[8] Lewis SA, Ivanov IE, Lee GH, Cowan NJ. Organization of microtubules in dendrites and axons is determined by a short hydrophobic zipper in microtubule-associated proteins MAP2 and tau. Nature. 1989;342(6249):498–505. Epub 1989/11/30. doi: 10.1038/342498a0 . 2511449 10.1038/342498a0

[9] Kanai Y, Chen J, Hirokawa N. Microtubule bundling by tau proteins in vivo: analysis of functional domains. The EMBO journal. 1992;11(11):3953–61. Epub 1992/11/01. ; 1396588 10.1002/j.1460-2075.1992.tb05489.x PMC556906

[10] Brandt R, Lee G. Functional organization of microtubule-associated protein tau. Identification of regions which affect microtubule growth, nucleation, and bundle formation in vitro. The Journal of biological chemistry. 1993;268(5):3414–9. Epub 1993/02/15. . 8429017

[11] Gustke N, Trinczek B, Biernat J, Mandelkow EM, Mandelkow E. Domains of tau protein and interactions with microtubules. Biochemistry. 1994;33(32):9511–22. Epub 1994/08/16. . 8068626 10.1021/bi00198a017

[12] Scott CW, Klika AB, Lo MM, Norris TE, Caputo CB. Tau protein induces bundling of microtubules in vitro: comparison of different tau isoforms and a tau protein fragment. Journal of neuroscience research. 1992;33(1):19–29. Epub 1992/09/01. doi: 10.1002/jnr.490330104 . 1360542 10.1002/jnr.490330104

[13] Chung PJ, Song C, Deek J, Miller HP, Li Y, Choi MC, et al Tau mediates microtubule bundle architectures mimicking fascicles of microtubules found in the axon initial segment. Nature communications. 2016;7:12278 Epub 2016/07/28. doi: 10.1038/ncomms12278 ; 27452526 10.1038/ncomms12278 PMC4962469

[14] Chapin SJ, Bulinski JC, Gundersen GG. Microtubule bundling in cells. Nature. 1991;349(6304):24 Epub 1991/01/03. doi: 10.1038/349024a0 . 1670738 10.1038/349024a0

[15] Lee G, Brandt R. Microtubule-bundling studies revisited: is there a role for MAPs? Trends in cell biology. 1992;2(10):286–9. Epub 1992/10/01. . 14731912 10.1016/0962-8924(92)90106-w

[16] Schiff PB, Horwitz SB. Taxol stabilizes microtubules in mouse fibroblast cells. Proceedings of the National Academy of Sciences of the United States of America. 1980;77(3):1561–5. Epub 1980/03/01. ; 6103535 10.1073/pnas.77.3.1561 PMC348536

[17] Wehland J, Sandoval IV. Cells injected with guanosine 5'-[alpha, beta-methylene]triphosphate, an alpha, beta-nonhydrolyzable analog of GTP, show anomalous patterns of tubulin polymerization affecting cell translocation, intracellular movement, and the organization of Golgi elements. Proceedings of the National Academy of Sciences of the United States of America. 1983;80(7):1938–41. Epub 1983/04/01. ; 6572952 10.1073/pnas.80.7.1938 PMC393726

[18] Bhattacharyya B, Sackett DL, Wolff J. Tubulin, hybrid dimers, and tubulin S. Stepwise charge reduction and polymerization. The Journal of biological chemistry. 1985;260(18):10208–16. Epub 1985/08/25. . 3894367

[19] Melki R, Kerjan P, Waller JP, Carlier MF, Pantaloni D. Interaction of microtubule-associated proteins with microtubules: yeast lysyl- and valyl-tRNA synthetases and tau 218–235 synthetic peptide as model systems. Biochemistry. 1991;30(49):11536–45. Epub 1991/12/10. . 1747372 10.1021/bi00113a008

[20] Walczak CE, Shaw SL. A MAP for bundling microtubules. Cell. 2010;142(3):364–7. Epub 2010/08/10. doi: 10.1016/j.cell.2010.07.023 . 20691897 10.1016/j.cell.2010.07.023

[21] Sato Y, Akitsu M, Amano Y, Yamashita K, Ide M, Shimada K, et al The novel PAR-1-binding protein MTCL1 has crucial roles in organizing microtubules in polarizing epithelial cells. Journal of cell science. 2013;126(Pt 20):4671–83. Epub 2013/08/02. doi: 10.1242/jcs.127845 . 23902687 10.1242/jcs.127845

[22] Sato Y, Hayashi K, Amano Y, Takahashi M, Yonemura S, Hayashi I, et al MTCL1 crosslinks and stabilizes non-centrosomal microtubules on the Golgi membrane. Nature communications. 2014;5:5266 Epub 2014/11/05. doi: 10.1038/ncomms6266 . 25366663 10.1038/ncomms6266

[23] Satake T, Yamashita K, Hayashi K, Miyatake S, Tamura-Nakano M, Doi H, et al MTCL1 plays an essential role in maintaining Purkinje neuron axon initial segment. The EMBO journal. 2017: e201695630. doi: 10.15252/embj.201695630 . 28283581 10.15252/embj.201695630 PMC5412768

[24] Yamashita K, Suzuki A, Satoh Y, Ide M, Amano Y, Masuda-Hirata M, et al The 8th and 9th tandem spectrin-like repeats of utrophin cooperatively form a functional unit to interact with polarity-regulating kinase PAR-1b. Biochemical and biophysical research communications. 2010;391(1):812–7. Epub 2009/12/01. doi: 10.1016/j.bbrc.2009.11.144 . 19945424 10.1016/j.bbrc.2009.11.144

[25] Masuda-Hirata M, Suzuki A, Amano Y, Yamashita K, Ide M, Yamanaka T, et al Intracellular polarity protein PAR-1 regulates extracellular laminin assembly by regulating the dystroglycan complex. Genes to cells: devoted to molecular & cellular mechanisms. 2009;14(7):835–50. Epub 2009/06/25. doi: 10.1111/j.1365-2443.2009.01315.x . 19549170 10.1111/j.1365-2443.2009.01315.x

[26] Lupas AN, Gruber M. The structure of alpha-helical coiled coils. Advances in protein chemistry. 2005;70:37–78. Epub 2005/04/20. doi: 10.1016/S0065-3233(05)70003-6 . 15837513 10.1016/S0065-3233(05)70003-6

[27] Mason JM, Arndt KM. Coiled coil domains: stability, specificity, and biological implications. Chembiochem: a European journal of chemical biology. 2004;5(2):170–6. Epub 2004/02/05. doi: 10.1002/cbic.200300781 . 14760737 10.1002/cbic.200300781

[28] Webster DR, Borisy GG. Microtubules are acetylated in domains that turn over slowly. Journal of cell science. 1989;92 (Pt 1):57–65. Epub 1989/01/01. . 2674164 10.1242/jcs.92.1.57

[29] Engelman DM, Xiao Zhou F, Cocco MJ, Russ WP, Brunger AT. Interhelical hydrogen bonding drives strong interactions in membrane proteins. Nature Structural Biology. 2000;7:154–60. doi: 10.1038/72430 . 10655619 10.1038/72430

[30] Meng F-G, Zeng X, Hong Y-K, Zhou H-M. Dissociation and unfolding of GCN4 leucine zipper in the presence of sodium dodecyl sulfate. Biochimie. 2001;83:953–6. doi: 10.1016/S0300-9084(01)01340-2 11728632 10.1016/s0300-9084(01)01340-2

[31] Pollard TD. Actin and Actin-Binding Proteins. Cold Spring Harbor Perspectives in Biology. 2016;8:a018226 doi: 10.1101/cshperspect.a018226 . 26988969 10.1101/cshperspect.a018226 PMC4968159

