# Supplementary material for: Molecular basis of the microtubule-regulating activity of microtubule crosslinking factor 1
Source: PLoS One. 2017 Aug 7;12(8):e0182641. doi: 10.1371/journal.pone.0182641 (PMC5546597; doi:10.1371/journal.pone.0182641)
Supplement: S1 Fig — Immunoprecipitation assays were performed using an anti-GFP antibody and extracts of HEK293T cells co-expressing GFP or GFP-tagged CC1 and RFP-tagged CC1. (PDF) [file pone.0182641.s001.pdf]

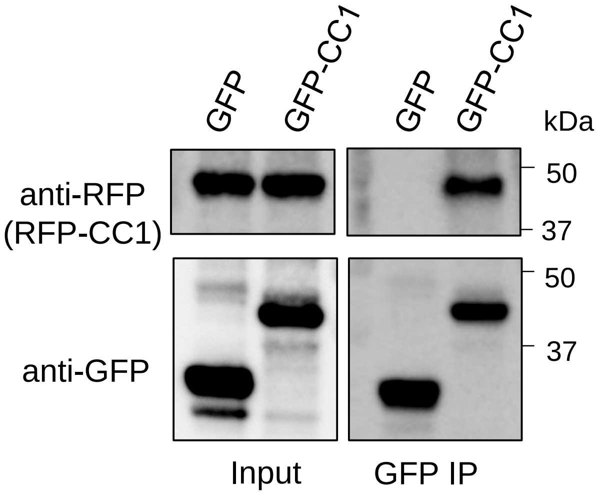

S1 Fig. CC1 shows a homo-interaction *in vivo*.

Immunoprecipitation assays were performed using an anti-GFP antibody and extracts of HEK293T cells co-expressing GFP or GFP-tagged CC1 and RFP-tagged CC1.
