# Supplementary material for: Molecular basis of the microtubule-regulating activity of microtubule crosslinking factor 1
Source: PLoS One. 2017 Aug 7;12(8):e0182641. doi: 10.1371/journal.pone.0182641 (PMC5546597; doi:10.1371/journal.pone.0182641)
Supplement: S2 Fig — (A) The amino acid sequence of mouse MTCL1 from 1 to 450 is shown. Red and green boxes indicate N-MTBD and the proline-rich region, respectively. Proline residues are red. The sequences corresponding to CC1 and CC2 are boxed by black, in which residues in the “d” position of the heptad repeat (a-b-c-d-e-f-g)n are blue. The amino acids in the first half of CC1 are bold, and the mutated residues are indicated by asterisks. Note that the heptad pattern of the first and latter half of CC1 is interrupted by a single residue, glutamate (red arrow). (B) Secondary structures of the first half of CC1 wt and its mutants were predicted by the PSIPRED program (http://bioinf.cs.ucl.ac.uk/psipred/). Positions of mutated leucine residues are indicated by red rectangles. Seven residue repeats, (a-b-c-d-e-f-g), are indicated by black brackets under the sequences. (PDF) [file pone.0182641.s002.pdf]

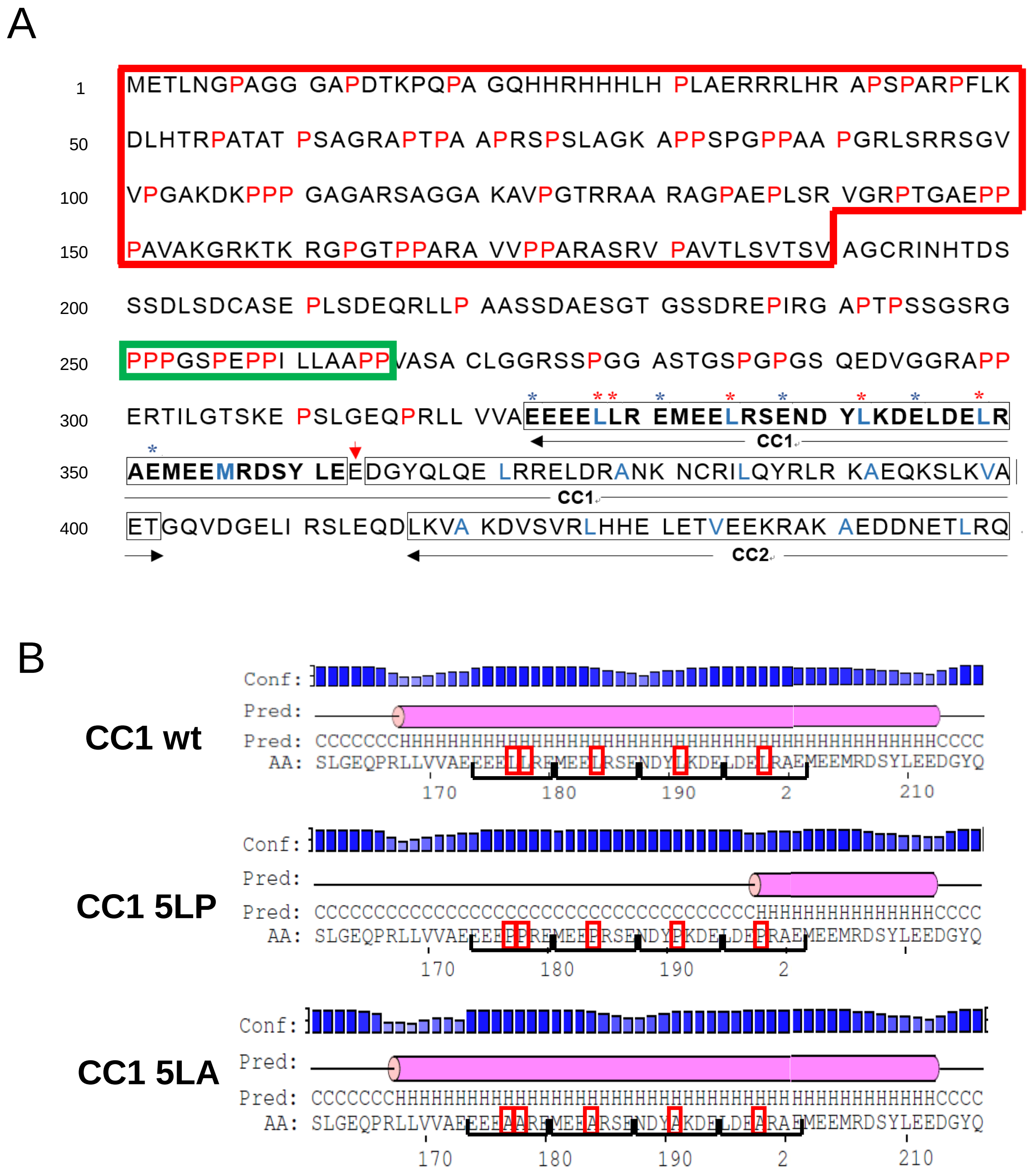

S2 Fig. The amino acid sequence of the MTCL1 N-terminal region and the predicted secondary structure of the first half of CC1.

(A) The amino acid sequence of mouse MTCL1 from 1 to 450 is shown. Red and green boxes indicate N-MTBD and the proline-rich region, respectively. Proline residues are red. The sequences corresponding to CC1 and CC2 are boxed by black, in which

residues in the "*d*" position of the heptad repeat (***a-b-c-d-e-f-g***)$_{\mathbf{n}}$ are blue. The amino acids in the first half of CC1 are bold, and the mutated residues are indicated by asterisks. Note that the heptad pattern of the first and latter half of CC1 is interrupted by a single residue, glutamate (red arrow). (B) Secondary structures of the first half of CC1 wt and its mutants were predicted by the PSIPRED program (http://bioinf.cs.ucl.ac.uk/psipred/). Positions of mutated leucine residues are indicated by red rectangles. Seven residue repeats, (***a-b-c-d-e-f-g***), are indicated by black brackets under the sequences.
