# Supplementary material for: Molecular basis of the microtubule-regulating activity of microtubule crosslinking factor 1
Source: PLoS One. 2017 Aug 7;12(8):e0182641. doi: 10.1371/journal.pone.0182641 (PMC5546597; doi:10.1371/journal.pone.0182641)
Supplement: S3 Fig — Immunostaining of V5 and α-tubulin in HeLa-K cells expressing V5-MTCL1 full wt and its mutant lacking C-MTBD (fullΔKR). Right panels show cells fixed after cold treatment on ice for 1 h. Scale bars, 10 μm. (PDF) [file pone.0182641.s003.pdf]

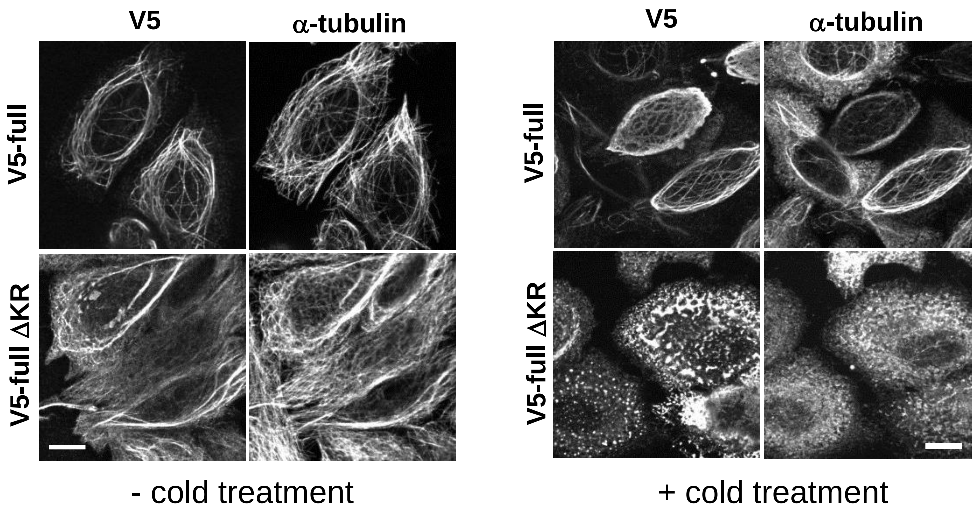

S3 Fig. MTCL1 cannot stabilize MTs without C-MTBD. Immunostaining of V5 and α-tubulin in HeLa-K cells expressing V5-MTCL1 full wt and its mutant lacking C-MTBD (fullΔKR). Right panels show cells fixed after cold treatment on ice for 1 h. Scale bars, 10 μm.
