# Supplementary material for: Molecular basis of the microtubule-regulating activity of microtubule crosslinking factor 1
Source: PLoS One. 2017 Aug 7;12(8):e0182641. doi: 10.1371/journal.pone.0182641 (PMC5546597; doi:10.1371/journal.pone.0182641)
Supplement: S4 Fig — (A) The CC1 fragment purified from E.coli (with a calculated molecular mass of ~10.5 kDa) was solubilized in SDS sample buffer containing 2% SDS with or without heat treatment (100°C, 10 min) and then subjected to SDS-PAGE analysis and CBB staining. (B) Crosslinking result of monomeric CC1 with the chemical crosslinking reagent DST. The purified CC1 fragment (0.52 mg/ml) dissolved in PBS was incubated for 30 min at 25°C in the presence of various concentrations of DST (lanes 2–4: 0.1, 0.3 and 0.9 mg/ml, respectively). The reaction was stopped by addition of a Tris-HCl stock solution (pH 7.5) at a final concentration of 50 mM followed by incubation for 15 min at 25°C.(C) Possible modes of MTCL1 assembly based on the CC1-CC1 homo-interaction. (PDF) [file pone.0182641.s004.pdf]

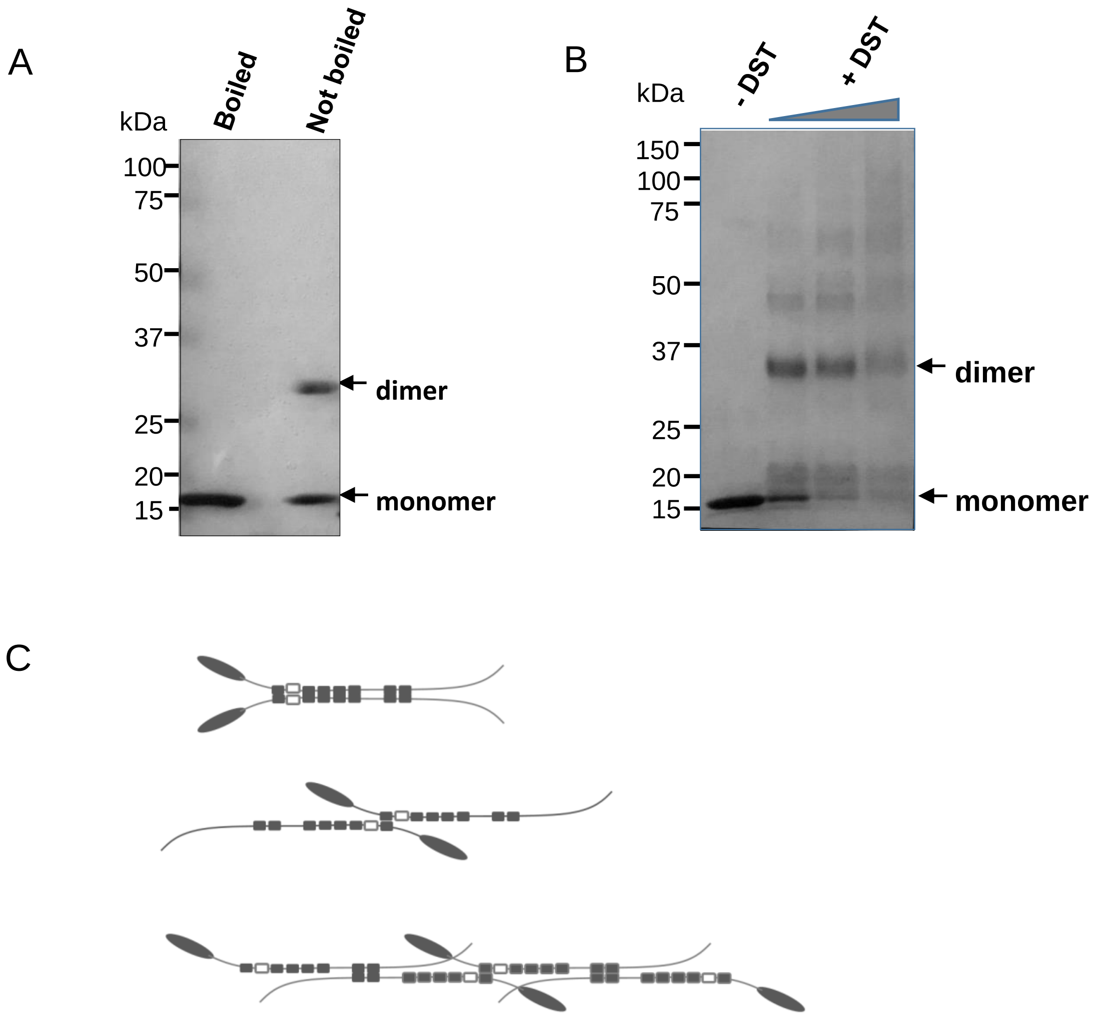

S4 Fig. CC1 forms a dimer (A) The CC1 fragment purified from *E.coli* (with a calculated molecular mass of ~10.5 kDa) was solubilized in SDS sample buffer containing 2% SDS with or without heat treatment (100°C, 10 min) and then subjected to SDS-PAGE analysis and CBB staining. (B) Crosslinking result of monomeric CC1 with the chemical crosslinking reagent DST. The purified CC1 fragment (0.52 mg/ml) dissolved in PBS was incubated for 30 min at 25°C in the presence of various concentrations of DST (lanes 2–4: 0.1, 0.3 and 0.9 mg/ml, respectively). The reaction was stopped by addition of a Tris-HCl stock solution (pH 7.5) at a final concentration of 50 mM followed by incubation for 15 min at 25°C. (C) Possible modes of MTCL1 assembly based on the CC1-CC1 homo-interaction.
