# Supplementary material for: Molecular basis of the microtubule-regulating activity of microtubule crosslinking factor 1
Source: PLoS One. 2017 Aug 7;12(8):e0182641. doi: 10.1371/journal.pone.0182641 (PMC5546597; doi:10.1371/journal.pone.0182641)
Supplement: S5 Fig — Streptavidin pull-down assays performed with extracts of HEK293T cells co-expressing the indicated MTCL1 mutants. (A) The C fragment interacts with itself, but not with the N fragment. (B) The N1 fragment interacts with itself, but not with the N6 fragment. (C–F) Immunoprecipitation assays by anti-GFP antibody examining interactions of RFP-CC3 (C), CC4 (D), CC5 (E) or CC6 (F) with the indicated GFP proteins. (PDF) [file pone.0182641.s005.pdf]

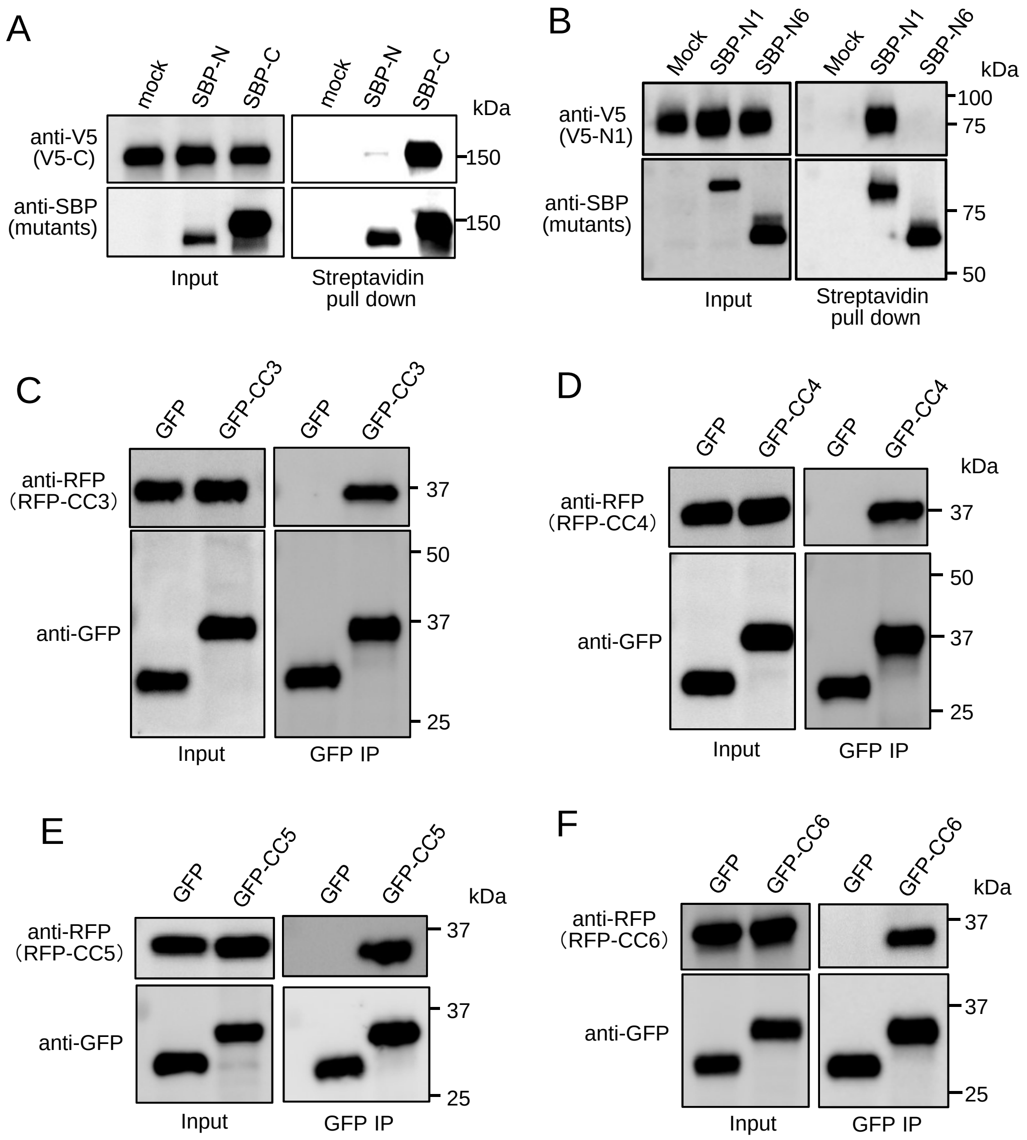

S5 Fig. Supplementary data for identification of homo-interaction regions in the MTCL1 N-terminus. Streptavidin pull-down assays performed with extracts of HEK293T cells co-expressing the indicated MTCL1 mutants. (A) The C fragment interacts with itself, but not with the N fragment. (B) The N1 fragment interacts with itself, but not with the N6 fragment. (C–F) Immunoprecipitation assays by anti-GFP antibody examining interactions of RFP-CC3 (C), CC4 (D), CC5 (E) or CC6 (F) with the indicated GFP proteins.
