# Supplementary material for: Molecular basis of the microtubule-regulating activity of microtubule crosslinking factor 1
Source: PLoS One. 2017 Aug 7;12(8):e0182641. doi: 10.1371/journal.pone.0182641 (PMC5546597; doi:10.1371/journal.pone.0182641)
Supplement: S6 Fig — Streptavidin pull-down assays performed with extracts of HEK293T cells co-expressing SBP-CC1 (A), CC5 (B) or CC6 (C) and the indicated GFP-fusion proteins of the N-terminal CCs. (PDF) [file pone.0182641.s006.pdf]

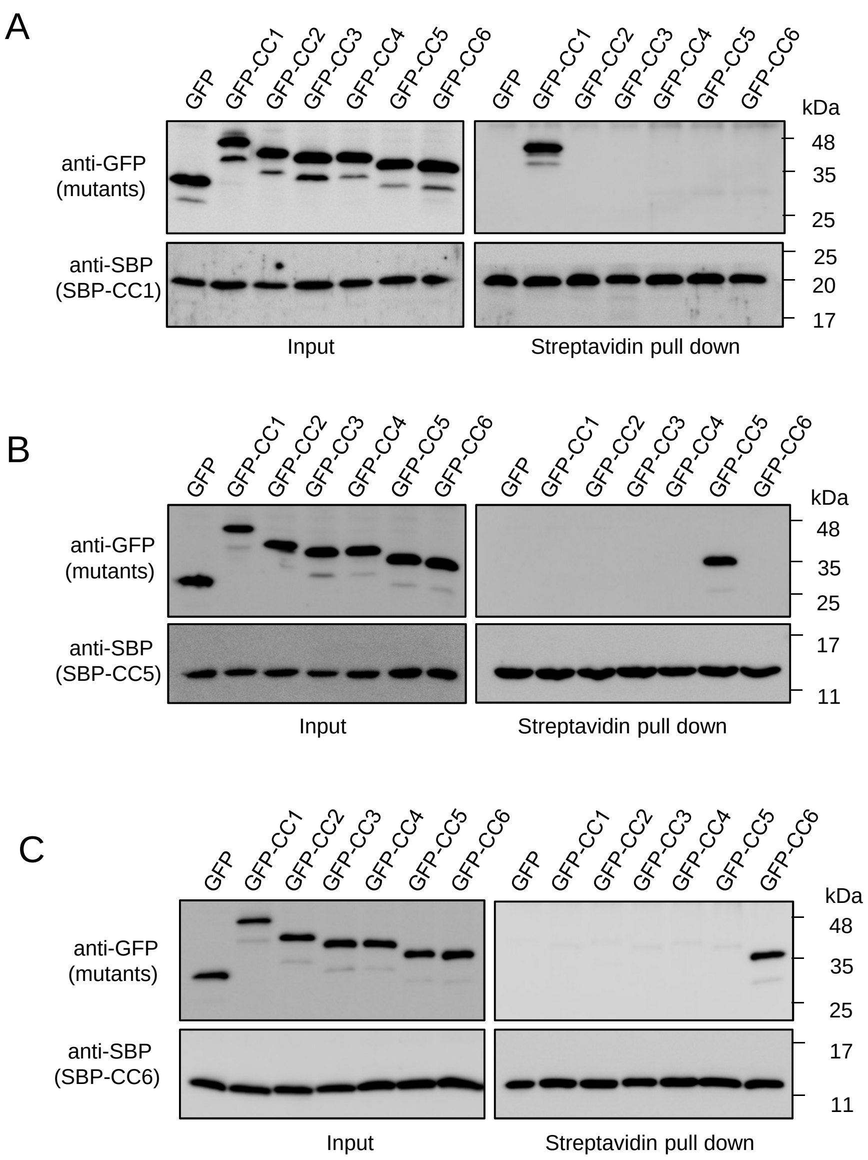

S6 Fig. N-terminal CCs show highly specific homo-interactions. Streptavidin pull-down assays performed with extracts of HEK293T cells co-expressing SBP-CC1 (A), CC5 (B) or CC6 (C) and the indicated GFP-fusion proteins of the N-terminal CCs.
