# Supplementary material for: Molecular basis of the microtubule-regulating activity of microtubule crosslinking factor 1
Source: PLoS One. 2017 Aug 7;12(8):e0182641. doi: 10.1371/journal.pone.0182641 (PMC5546597; doi:10.1371/journal.pone.0182641)
Supplement: S7 Fig — (A) Streptavidin pull-down assays performed with extracts of HEK293T cells co-expressing SBP-tagged mutants and V5-tagged C1. (B) Alignment of the amino acid sequence of C9. (C) GST pull-down assays performed with a mixture of purified C9 fragment and GST or GST-C9. (PDF) [file pone.0182641.s007.pdf]

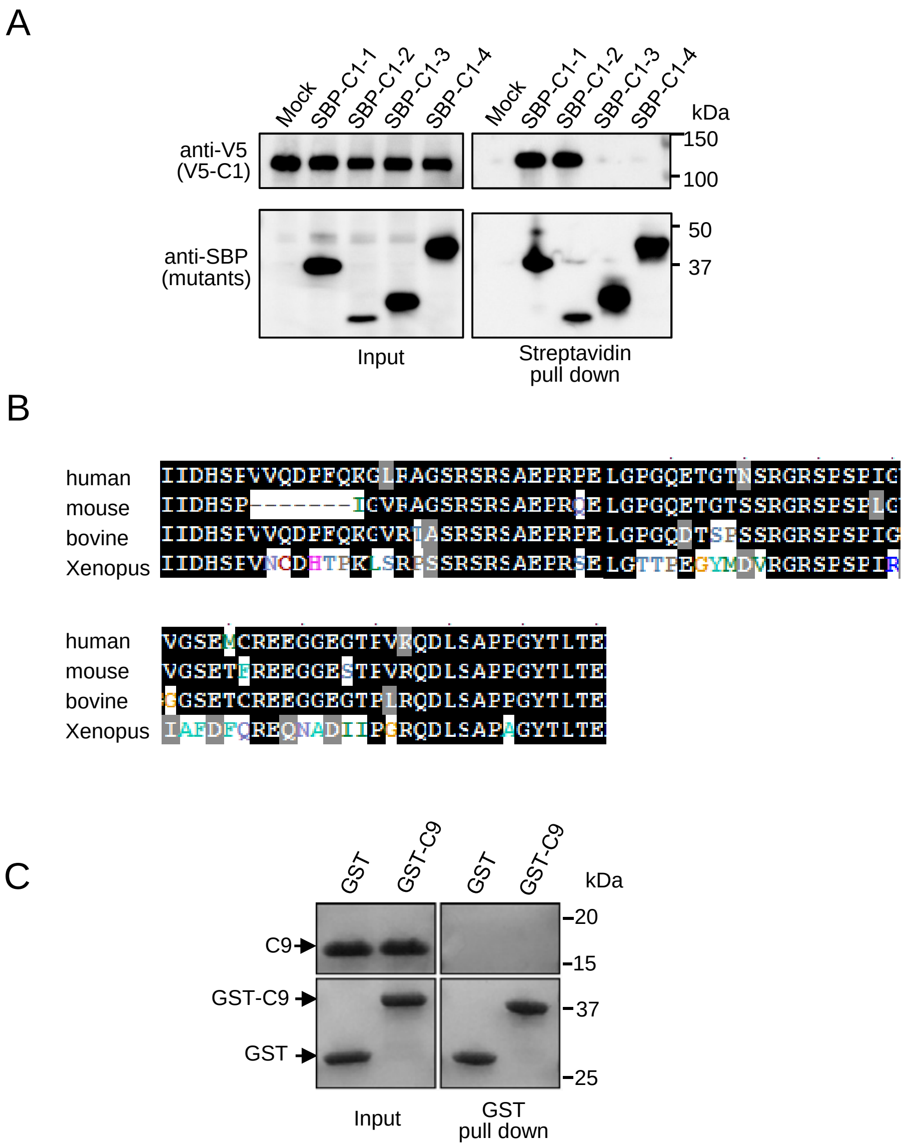


**S7 Fig. Supplementary data for identification of homo-interaction regions in the MTCL1 C-terminus.** (A) Streptavidin pull-down assays performed with extracts of HEK293T cells co-expressing SBP-tagged mutants and V5-tagged C1. (B) Alignment of the amino acid sequence of C9. (C) GST pull-down assays performed with a mixture of purified C9 fragment and GST or GST-C9.
