# Supplementary material for: Molecular basis of the microtubule-regulating activity of microtubule crosslinking factor 1
Source: PLoS One. 2017 Aug 7;12(8):e0182641. doi: 10.1371/journal.pone.0182641 (PMC5546597; doi:10.1371/journal.pone.0182641)
Supplement: S8 Fig — Immunostaining of V5 and α-tubulin in HeLa-K cells expressing V5-NCC3 wt and its 5LP mutant. Scale bars, 10 μm. (PDF) [file pone.0182641.s008.pdf]

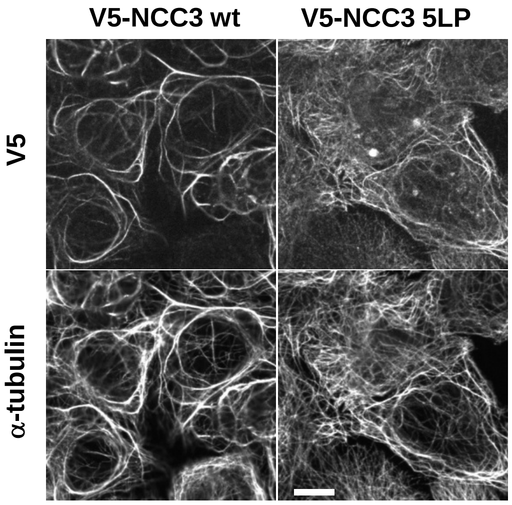

S8 Fig. Effect of the 5LP mutation in CC1 on the MT-crosslinking activity of V5-NCC3. Immunostaining of V5 and α-tubulin in HeLa-K cells expressing V5-NCC3 wt and its 5LP mutant. Scale bars, 10 μm.
