# Supplementary material for: Molecular basis of the microtubule-regulating activity of microtubule crosslinking factor 1
Source: PLoS One. 2017 Aug 7;12(8):e0182641. doi: 10.1371/journal.pone.0182641 (PMC5546597; doi:10.1371/journal.pone.0182641)
Supplement: S9 Fig — Hela-K cells were triply immunostained with antibodies against MTCL1, α-tubulin and acetylated tubulin, and analyzed by super-resolution microscopy. The bottom panels correspond to enlarged views of the rectangle region indicated in the top panels. Arrowheads indicate MTCL1 signals on MTs which are not strongly stained with anti-acetylated tubulin antibody. Scale bars, 5 μm (top panels) or 2 μm (bottom panels). (PDF) [file pone.0182641.s009.pdf]

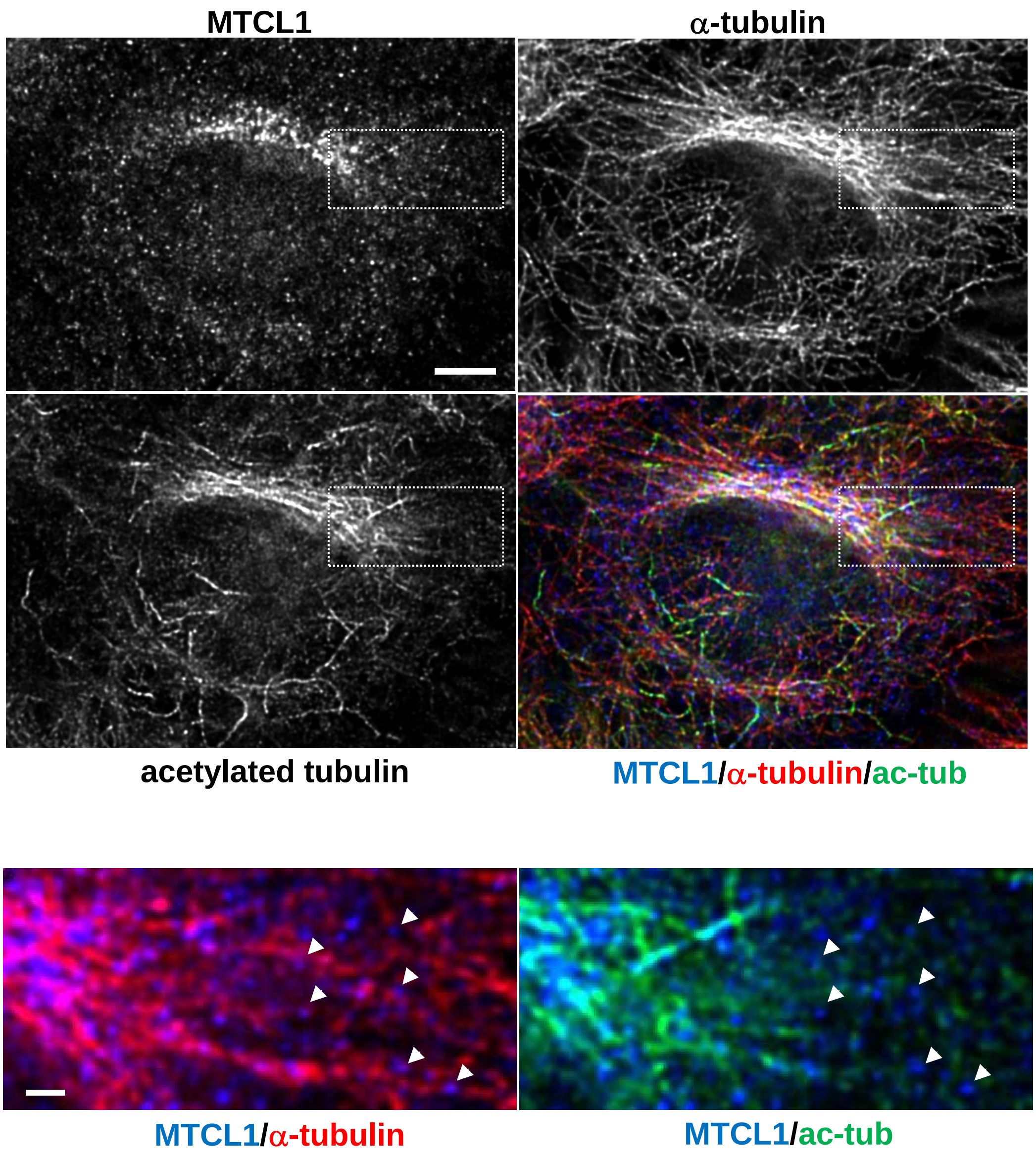

S9 Fig. MTs decorated by MTCL1 do not necessarily correspond to stabilized MTs. Hela-K cells were triply immunostained with antibodies against MTCL1, α-tubulin and acetylated tubulin, and analyzed by super-resolution microscopy. The bottom panels correspond to enlarged views of the rectangle region indicated in the top panels. Arrowheads indicate MTCL1 signals on MTs which are not strongly stained with anti-acetylated tubulin antibody. Scale bars, 5 µm (top panels) or 2 µm (bottom panels).
