# Supplementary material for: Molecular basis of the microtubule-regulating activity of microtubule crosslinking factor 1
Source: PLoS One. 2017 Aug 7;12(8):e0182641. doi: 10.1371/journal.pone.0182641 (PMC5546597; doi:10.1371/journal.pone.0182641)
Supplement: S1 Table — (PDF) [file pone.0182641.s010.pdf]

Amino acid numbers of mouse MTCL1 covered by each mutants

name	amino acid number
N	1-916
N1	1-508
N2	1-366
N3	209-515
N4	1-241
N6	491-916
N8	721-826
N9	827-916
NCC1	1-432
NCC3	1-548
NCC4	1-607
CC1+CC2	318-487
CC3+CC4	491-607
CC5+CC6	619-720
CC1	318-406
CC2	408-487
CC3	491-548
CC4	540-611
CC5	619-670
CC6	672-720

name	amino acid number
C	917-1945
C1	916-1680
C1-1	916-1133
C1-2	1134-1270
C1-3	1271-1415
C1-4	1416-1680
C2	1508-1945
C3	1583-1945
C4	1680-1945
C5	1767-1945
C2ΔC5	1508-1766
C6	1762-1861
C7	1850-1945
C8	1819-1858
C9	1762-1834
